# Discovery and preclinical evaluation of ZLC853 as a potential clinical candidate covalently targeting CDK12/13 kinases

**DOI:** 10.1016/j.apsb.2026.07.041

**Published:** 2026-07-29

**Authors:** Kaijie Zhou, Licheng Zhou, Jean C. Tien, Weixue Huang, Wenyan Liu, Xinyu Sun, Bohai Fan, Xin Huang, Jun Wang, Yu Chang, Jianzhang Yang, Yue Yin, Chen Su, Qingrun Li, Rahul Mannan, Somnath Mahapatra, Yunhui Cheng, Abigail J. Todd, Christina E. Wheeler, Shuqin Li, Yang Zhou, Xiaomei Ren, George Xiaoju Wang, Zhen Wang, Arul M. Chinnaiyan, Ke Ding

**Affiliations:** a State Key Laboratory of Chemical Biology, Shanghai Institute of Organic Chemistry, Chinese Academy of Sciences, Shanghai 200032, China; b University of Chinese Academy of Sciences, Beijing 101408, China; c International Cooperative Laboratory of Traditional Chinese Medicine Modernization and Innovative Drug Discovery of Chinese Ministry of Education (MOE), Guangzhou City Key Laboratory of Precision Chemical Drug Development, College of Pharmacy, Jinan University, Guangzhou 511400, China; d Michigan Center for Translational Pathology, University of Michigan, Ann Arbor, MI 48109, USA; e Department of Pathology, University of Michigan, Ann Arbor, MI 48109, USA; f National Facility for Protein Science in Shanghai, Shanghai Advanced Research Institute, Chinese Academy of Science, Shanghai 201210, China; g Howard Hughes Medical Institute, University of Michigan, Ann Arbor, MI 48109, USA; h Rogel Cancer Center, University of Michigan, Ann Arbor, MI 48109, USA; i Department of Urology, University of Michigan, Ann Arbor, MI 48109, USA

**Keywords:** CDK12, CDK13, Covalent inhibitor, Rational design, Oral bioavailability, Crystal structure, Prostate cancer, Potential clinical candidate

## Abstract

Previous studies highlight the therapeutic promise of inhibiting cyclin-dependent kinases 12 and 13 (CDK12/13) for castration-resistant prostate cancer (CRPC) and other malignancies. However, developing small molecule modulators of CDK12/13 that exhibit an optimal combination of potency, selectivity and favorable pharmacokinetics (PK) remains challenging. In this study, we present **ZLC853**, a potent, selective, and orally bioavailable covalent inhibitor of CDK12/13 developed through PK-oriented optimization of our previously reported inhibitor YJZ5118. **ZLC853** potently inhibited CDK12 and CDK13 with IC_50_ values of 40.0 and 18.2 nmol/L, respectively, and displayed exceptional kinase selectivity in a comprehensive KinomeScan assay. Its covalent binding mechanism was unequivocally validated through mass spectrometry, co-crystal structure analysis, cellular pull-down assays, and washout experiments. Functionally, **ZLC853** suppressed the transcription of DNA damage response genes, induced DNA damage, and promoted apoptosis. Additionally, **ZLC853** exhibited good oral bioavailability and robust antitumor activity across multiple prostate cancer models, both *in vitro* and *in vivo*, supporting it as a promising candidate for clinical investigation.

## Graphical abstract

PK-oriented optimization afforded **ZLC853**, a potent, selective, and orally bioavailable covalent inhibitor of CDK12/13, representing a promising candidate for clinical CDK12/13-targeted cancer therapy.

**Figure:**
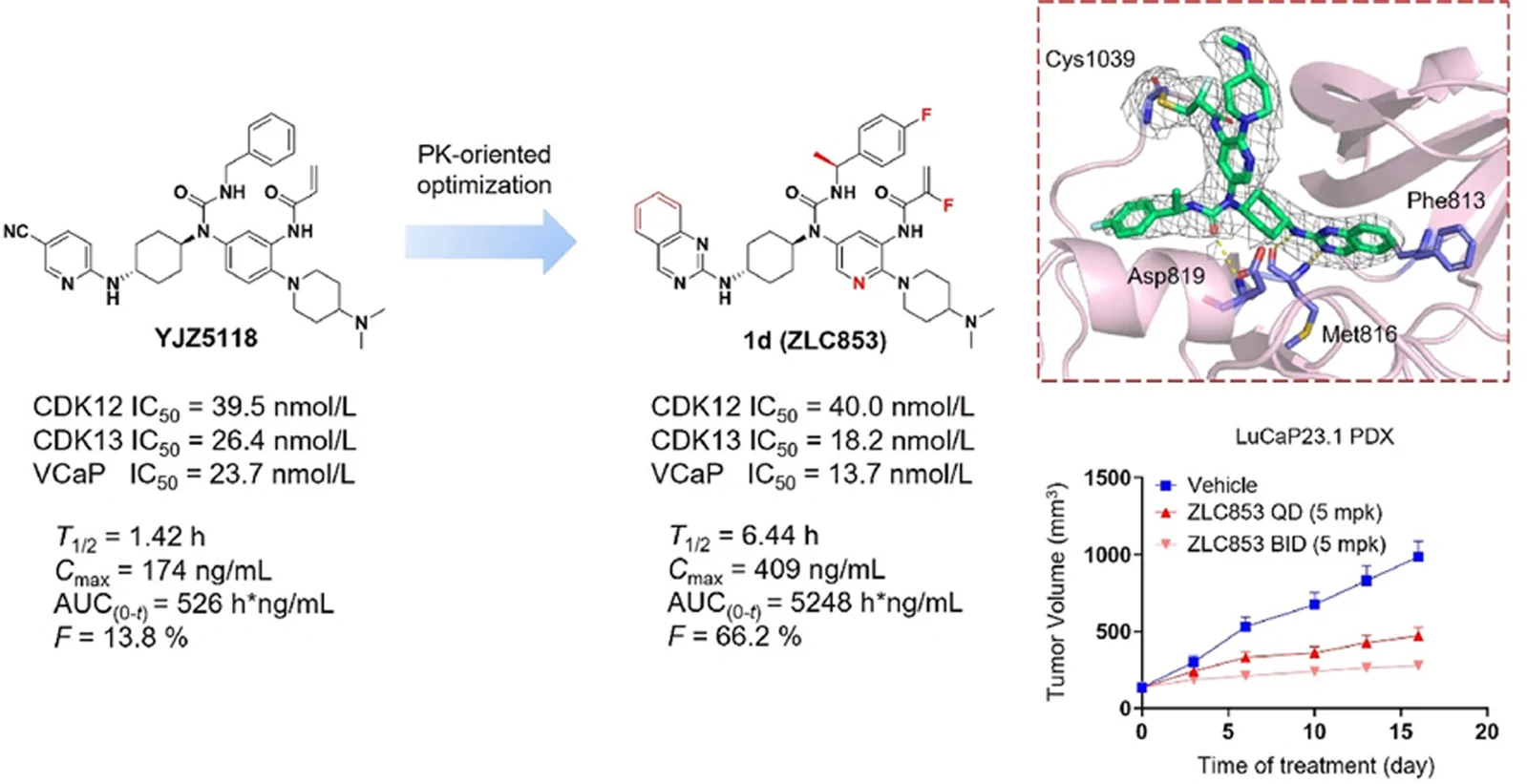

## 1 Introduction

Transcription-associated cyclin-dependent kinase 12 (CDK12) and its paralog CDK13 are serine/threonine kinases that share 92% homology within their kinase domains^1^. Upon activation through complex formation with cyclin K (CCNK), CDK12 and CDK13 phosphorylate the C-terminal domain (CTD) of RNA polymerase II (RNA Pol II), thereby playing key regulatory roles in transcriptional elongation, termination, splicing, cleavage, and polyadenylation^2^, ^3^, ^4^. These kinases are crucial for maintaining genomic stability through the DNA damage response (DDR). However, their aberrant activity is linked to oncogenesis in multiple cancer types^5^, ^6^, ^7^, ^8^. Our previous studies identified CDK12 mutations in 4.7%–6.9% of patients with metastatic castration-resistant prostate cancer (mCRPC)^6^. Genetic knockdown or pharmacological inhibition—including targeted degradation—of CDK12/13 resulted in suppressed transcription of DDR genes, accumulation of DNA damage, induction of apoptosis, and significant inhibition of prostate cancer cell proliferation *in vitro* and *in vivo*^9^, ^10^, ^11^, ^12^, ^13^. These findings highlight CDK12/13 as promising therapeutic targets for the treatment of prostate cancer.

Substantial efforts have been devoted to developing modulators of CDK12/13, leading to the discovery of orally bioavailable proteolysis-targeting chimera (PROTAC) degraders^10^, ^11^, ^12^^,^^14^^,^^15^ and cyclin K-directed molecular glues^16^, ^17^, ^18^, ^19^, ^20^, ^21^, ^22^, ^23^. Notably, CT7439, a cyclin K molecular glue degrader, has been advanced into clinical trials^22^^,^^23^. Whereas, although various highly potent covalent kinase inhibitors of CDK12/13 were discovered^13^^,^^24^, ^25^, ^26^, ^27^, ^28^, their development was impeded by the relatively low target specificity and/or poor pharmacokinetics (PK) properties (Fig. 1). Herein, we present the discovery of **ZLC853** as a potent, selective and orally bioavailable covalent inhibitor of CDK12/13. **ZLC853** potently inhibited CDK12 and CDK13 with IC_50_ values of 40.0 and 18.2 nmol/L, respectively, and demonstrated high selectivity over other CDK family members. Moreover, this compound exhibited compelling *in vivo* efficacy in multiple CRPC xenograft mouse models following oral administration, supporting it as a promising candidate for clinical development.

**Figure 1:**
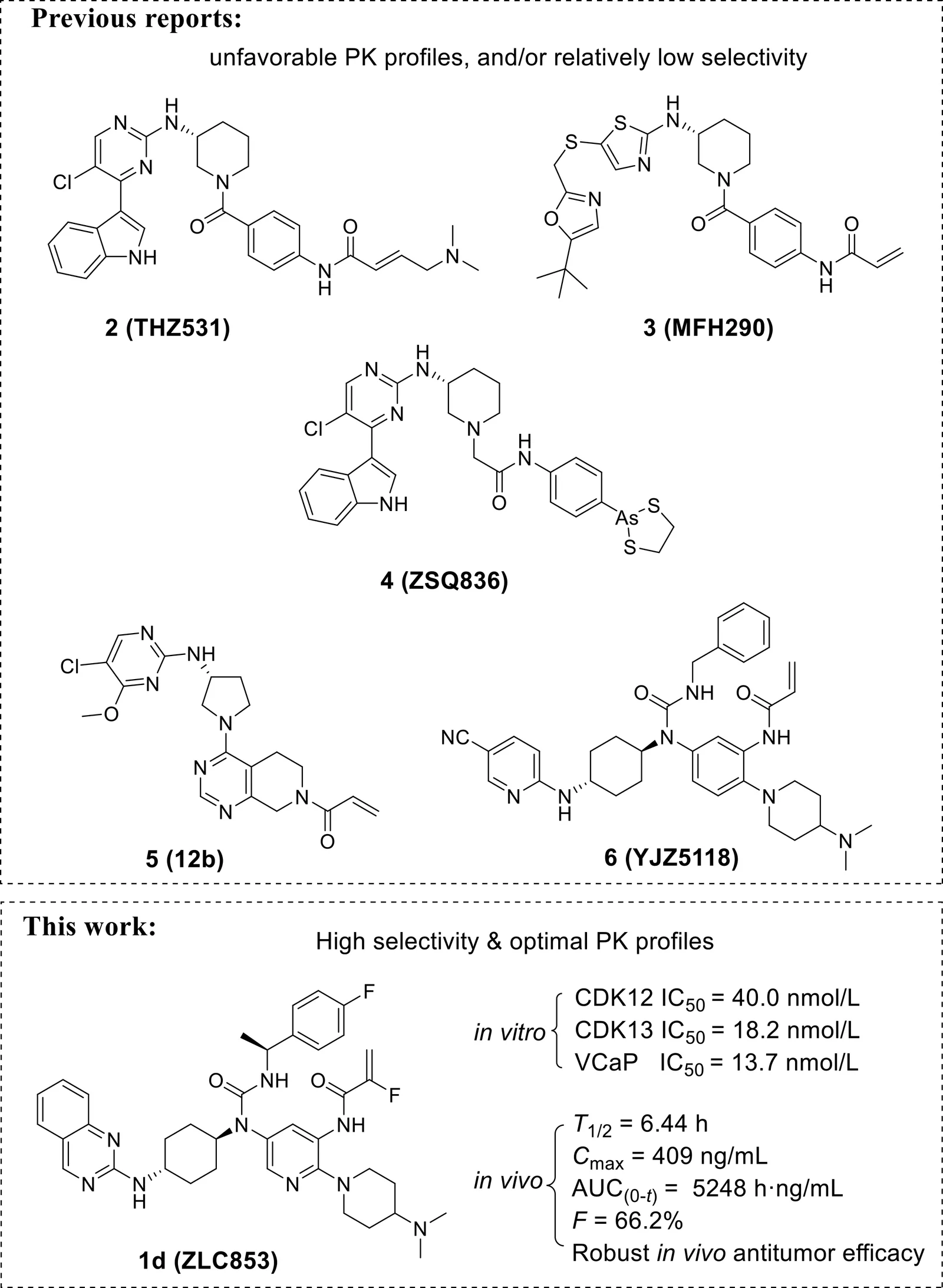
Progress on the discovery of covalent CDK12/13 inhibitors.

## 2 Results

### 2.1 Rational discovery of **ZLC853** as a new CDK12/13 covalent inhibitor

Applying a chemical adaptation approach^29^, we previously discovered YJZ5118 that effectively inhibited CDK12 and CDK13 with IC_50_ values of 39.5 and 26.4 nmol/L, respectively, and demonstrated high selectivity over other CDKs^13^. However, the compound exhibited suboptimal PK properties, showing a relatively low oral bioavailability of 13.8% and a short *T*_1/2_ value of 1.42 h in rats (Table 1). Structural feature analysis of YJZ5118 identified several metabolically vulnerable sites, including an unsubstituted benzyl group, the electron-rich phenyl group, and the reactive acrylamide moiety (Supporting Information Fig. S1)^30^, ^31^, ^32^, ^33^. Guided by these insights, we undertook structural optimization to develop new CDK12/13 inhibitors with improved PK profiles. The kinase inhibitory activities against CDK12 and CDK13 were evaluated using an ADP-Glo kinase assay, and their antiproliferative effects were assessed in VCaP prostate cancer cells, which are sensitive to CDK12/13 inhibition.

**Table 1:** PK profiles of compound YJZ5118 and **ZLC853** in rats.

Parameter	YJZ5118		ZLC853	
	i.v. 2 mg/kg	*p.o.* 10 mg/kg	i.v. 2 mg/kg	*p.o.* 10 mg/kg
*T*_1/2_ (h)	1.83	1.42	4.07	6.44
*T*_max_ (h)	0.08	2.33	0.08	4.67
*C*_max_ (ng/mL)	1107	174	516	409
AUC_0–*t*_ (h∗ng/mL)	772	526	1585	5248
AUC_0–∞_ (h∗ng/mL)	784	542	1606	5946
CL (mL/min/kg)	42.6	/	20.8	/
MRT_0–*t*_ (h)	0.98	2.84	4.30	7.65
MRT_0–∞_(h)	1.13	3.07	4.63	10.20
*F* (%)	13.8		66.2	

We first designed and synthesized compound **1a**, replacing the 3-cyanopyridine moiety with quinazoline based on the cocrystal structure of YJZ5118–CDK12 complex (PDB: 9JK1), which indicated additional space in the back pocket of the hinge region that could accommodate a larger group (Fig. 2). Compound **1a** retained potent inhibition of CDK12 and CDK13, with IC_50_ values of 25.0 and 22.4 nmol/L, respectively (Fig. 3). Notably, it exhibited a 5-fold increase in potency against VCaP cell growth compared to YJZ5118, likely attributable to the improved cellular membrane permeability resulting from the hydrophobic quinazoline group^34^. Unsubstituted benzyl group is a metabolically labile moiety, and introducing a methyl group at the methylene unit has been practiced to improve stability by prohibiting the potential dealkylation or oxidation^35^^,^^36^. Thus, diastereomers **1b** and **1c** were subsequently designed and synthesized. Notably, a nitrogen atom was also introduced into the oxidation-prone electron-rich phenyl group to potentially further improve the metabolic stability^37^. It was shown that the *S*-configured derivative (**1c**) maintained potent activity on both CDK12/13 kinases and VCaP cell proliferation compared to **1a**, while the *R*-configured methyl analog (**1b**) was two- to 3-fold less potent. Since the Michael acceptor warhead can react with off-target nucleophiles, fine-tuning its electrophilicity is essential to balancing activity, selectivity and PK properties^38^. *α*-Fluoro acrylamide has been well profiled to be advantageous over the unmol/Lodified acrylamide, by improving lipophilicity, membrane permeability and stability, and decreasing the reaction rate with cysteine^39^. We thus incorporated *α*-fluoro acrylamide warhead to the molecule to yield compound **1d** (**ZLC853**), in which an additional fluoro-atom was also introduced at the *para*-position of the benzyl moiety to alleviate its metabolic liability. It was shown that **ZLC853** potently inhibited CDK12 (IC_50_ = 40.0 nmol/L), CDK13 (IC_50_ = 18.2 nmol/L), and VCaP cell proliferation (IC_50_ = 13.7 nmol/L) (Fig. 3).

**Figure 2:**
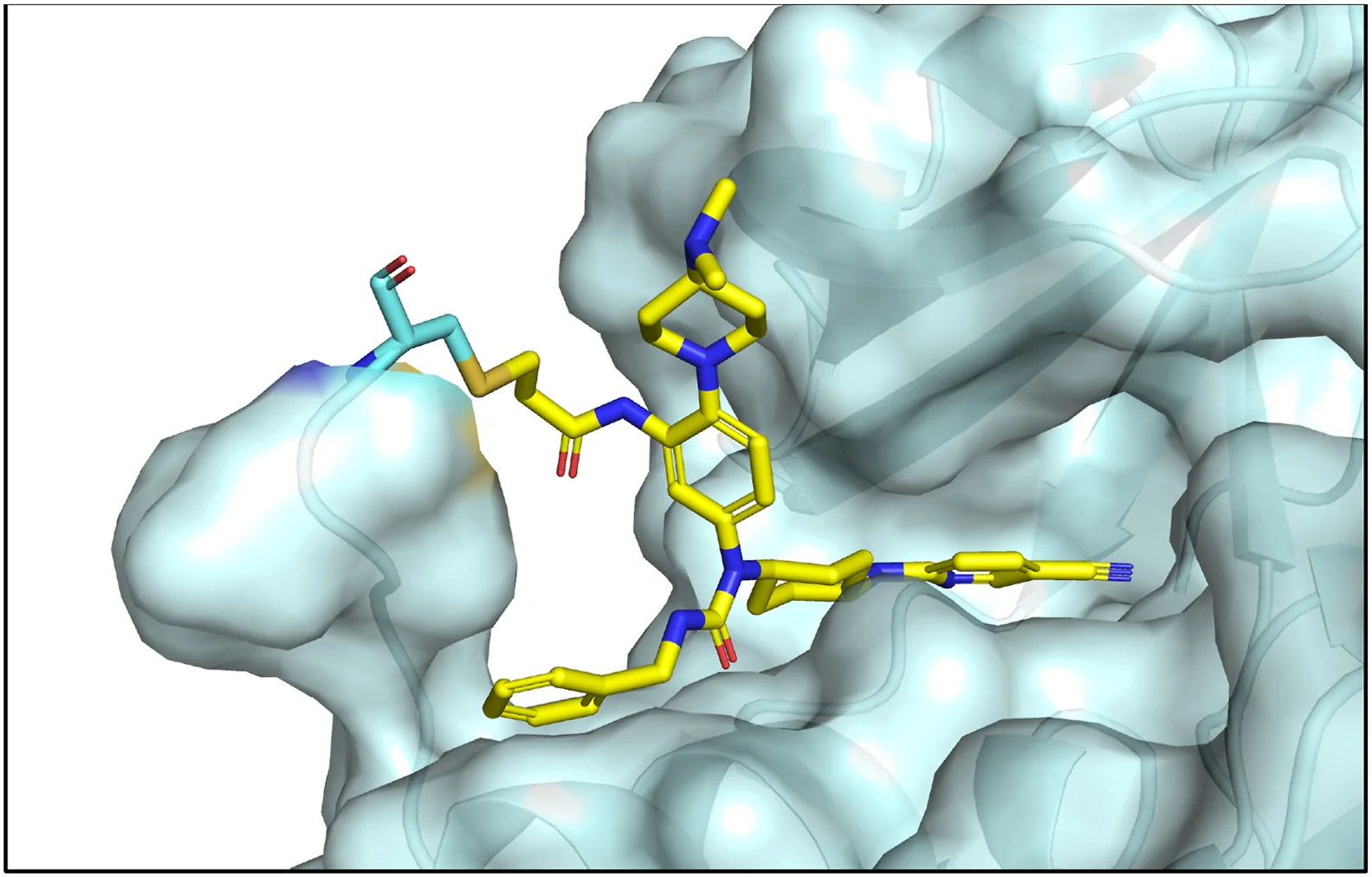
Co-crystal structure of CDK12/CCNK bound to compound YJZ5118 (PDB: 9JK1). CDK12 is shown in cyan surface, and YJZ5118 and C1039 of CDK12 are shown as yellow and cyan sticks, respectively.

**Figure 3:**
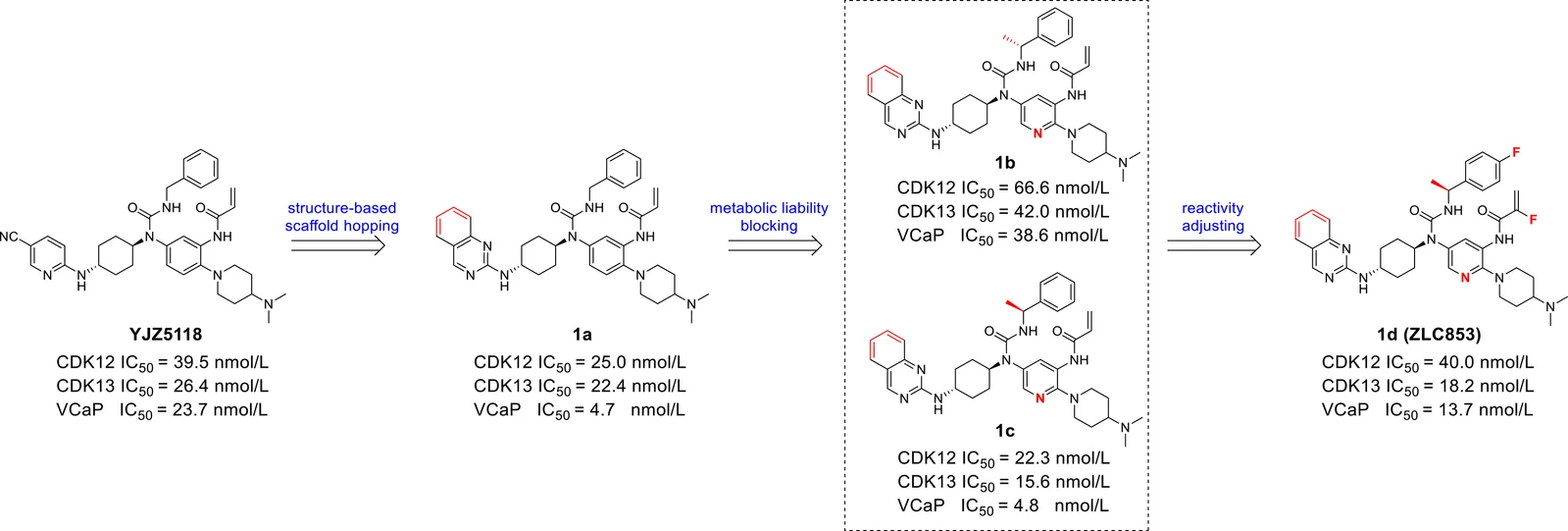
Structure-based discovery of **ZLC853** as a new orally bioavailable covalent CDK12/13 inhibitor.

### 2.2 Chemical synthesis of new CDK12/13 inhibitors

The designed molecules were synthesized by following procedures outlined in Scheme 1. The nucleophilic substitution reaction of compounds **2a**–**b** with *N*,*N*-dimethylpiperidin-4-amine provided compounds **3a**–**b**, which further reacted with *trans*-*N*-Boc-1,4-cyclohexanedimine to offer intermediate **4a**–**b**. Deprotection of *tert*-butoxycarbonyl group of compounds **4a**–**b** and subsequent nucleophilic substitution reaction with 2-chloroquinazoline provided compounds **5a**–**b**. Compounds **6a**–**d** were synthesized by the nucleophilic substitution reaction between compounds **5a**–**b** with various isocyanates or carbamates. Reducing reaction of compounds **6a**–**d** provided compounds **7a**–**d**, which further reacted with various acyl chlorides to yield compounds **1a**–**d** and **ZLC1178**.

**Scheme 1:**
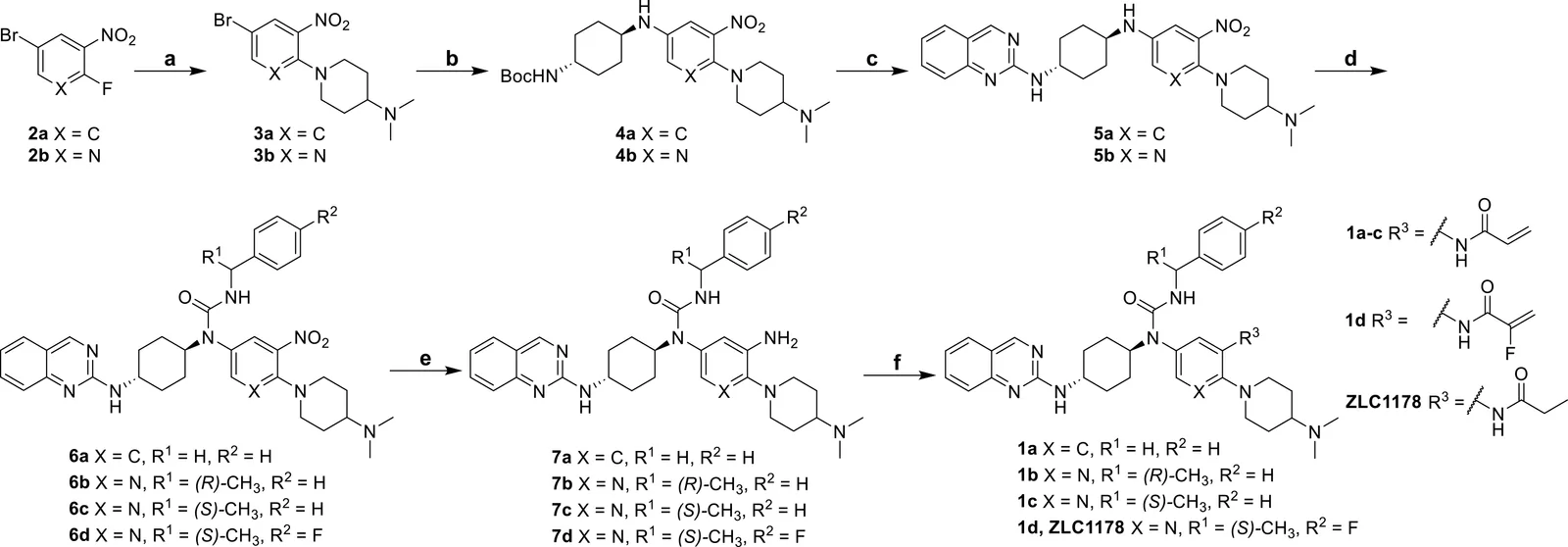
Synthesis of compound **1a-1d** and **ZLC1178**. Reagents and conditions: (a) *N*,*N*-dimethylpiperidin-4-amine, Cs_2_CO_3_, CH_3_CN, 60 °C, 12 h; (b) *trans*-*N*-Boc-1,4-cyclohexanedimine, cuprous iodide, K_3_PO_4_, d-proline, 100 °C, 12 h; (c) (i) TFA, CH_2_Cl_2_, rt, 1 h; (ii) 2-chloroquinazoline, Cs_2_CO_3_, DMF, 60 °C, 6 h; (d) **6a**–**c**: isocyanates, DIPEA, DMF, 90 °C, 16 h; **6d**: (i) *(S)*-1-(4-fluorophenyl) ethanamine, 4-nitrophenyl chloroformate, Et_3_N, THF, rt, 2 h; (ii) DIPEA, DMAP, DMF, 90 °C, 16 h; (e) hypodiboric acid, 4,4′-bipyridine, DMF, rt, 0.5 h; (f) **1a**–**c**, **ZLC1178**: acyl chloride, DIPEA, 0 °C to rt; **1d**: (i) 2-fluoracrylic acid, oxalyl chloride, CH_2_Cl_2_, 0 °C, ; (ii) DIPEA, CH_2_Cl_2_, 0 °C to rt.

The synthesis of compound **ZLC1223** is shown in Scheme 2. The nucleophilic substitution reaction of compound **9** with *trans*-*N*-Boc-1,4-cyclohexanedimine yielded intermediates **10**. Deprotection of *tert*-butoxycarbonyl group of compound **10** provided compound **11**. Compound **12** was prepared by nucleophilic substitution reaction of compound **2b** with 4-*N*-Boc-4-*N*-methyl-aminopiperidine, which further reacted with intermediate **11** to offer intermediate **13**. Compound **16** was prepared by a procedure similar to that used for **1d**. Deprotection of *tert*-butoxycarbonyl group of compound **16** and subsequent condensation reaction with carboxylic acid provided compound **ZLC1223**.

**Scheme 2:**
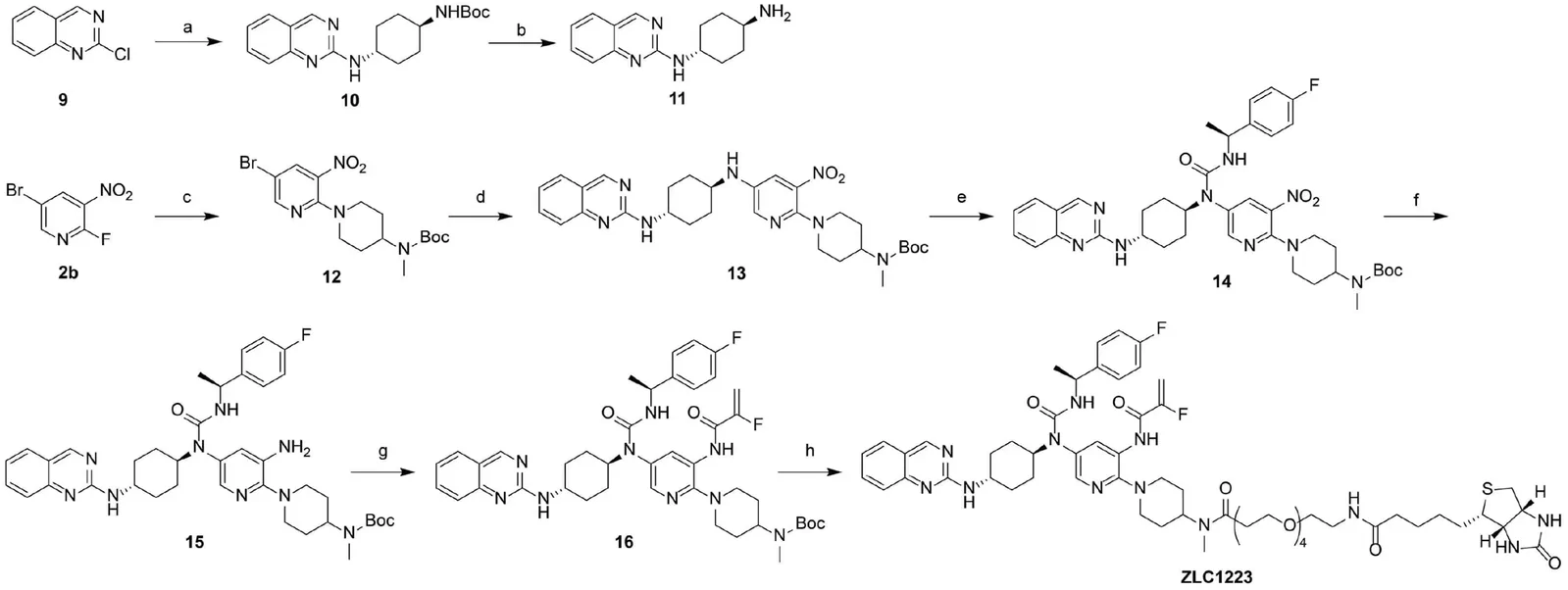
Synthesis of the compound **ZLC1223**. Eagents and conditions: (a) *trans*-*N*-Boc-1,4-cyclohexanedimine, Cs_2_CO_3_, DMF, 60 °C, 6 h; (b) TFA, CH_2_Cl_2_, rt, 1 h; (c) 4-*N*-Boc-4-*N*-methyl-aminopiperidine, Cs_2_CO_3_, CH_3_CN, 60 °C, 12 h; (d) compound **11**, Pd_2_ (dba)_3_, Brettphos, *tert*-BuONa, toluene, 100 °C, 12 h; (e) (i) *(S)*-1-(4-fluorophenyl) ethanamine, 4-nitrophenyl chloroformate, Et_3_N, THF, rt, 2 h; (ii) DIPEA, DMAP, DMF, 90 °C, 16 h; (f) hypodiboric acid, 4,4′-bipyridine, DMF, rt, 0.5 h; (g) (i) 2-fluoracrylic acid, oxalyl chloride, CH_2_Cl_2_, 0 °C; (ii) DIPEA, CH_2_Cl_2_, 0 °C to rt; (h) (i) TFA, CH_2_Cl_2_, rt, 1 h; (ii) HATU, DIPEA, DMF, rt, 1 h.

### 2.3 **ZLC853** covalently binds to CDK12 and CDK13

To confirm the covalent binding mode between **ZLC853** and CDK12/13, we incubated the CDK12/Cyclin K or CDK13/Cyclin K protein complex with a five-fold molar excess of **ZLC853** overnight at room temperature, followed by liquid chromatograph–mass spectrometry (LC–MS) analysis. A mass shift corresponding to the addition of one **ZLC853** molecule to CDK12 or CDK13 was detected (Fig. 4A and B), while Cyclin K remained unmol/Lodified (Supporting Information Fig. S2A and S2B). Capillary electrophoresis–MS/MS evaluation further indicated that the irreversible modification sites were Cys 1039 in CDK12 and Cys 1017 in CDK13 (Fig. S2C and S2D). The covalent binding of **ZLC853** to CDK12 was further elucidated by a co-crystal structure resolved at 3.28 Å. Similar to that of YJZ5118, the quinazolin-2-amine moiety of **ZLC853** formed two hydrogen bonds with the hinge residue Met 816, and the urea group formed an additional hydrogen bond with Asp 819. Continuous electron density clearly showed a covalent bond between the 2-fluoroacrylamide and Cys1039 (Fig. 4C and Table S1). To validate covalent engagement in a cellular context, we synthesized compound **ZLC1223**, a biotinylated analog of **ZLC853**, and **ZLC1178**, a reversible counterpart of **ZLC853**, respectively, for pull-down assays (Fig. 4D). As shown in Fig. 4E, **ZLC1223** effectively pulled down CDK12. Pre-treatment of VCaP cells with **ZLC853**—but not with the negative control compound **ZLC1178**—blocked this interaction, confirming target-specific covalent binding. We further evaluated the sustained inhibition of RNA pol II phosphorylation using a compound washout experiment. **ZLC853** maintained suppression of RNA pol II pSer 2 levels even at 16 h after washout, whereas **ZLC1178** showed no such effect (Fig. 4F). Collectively, these results demonstrate that **ZLC853** covalently binds with CDK12 and CDK13.

**Figure 4:**
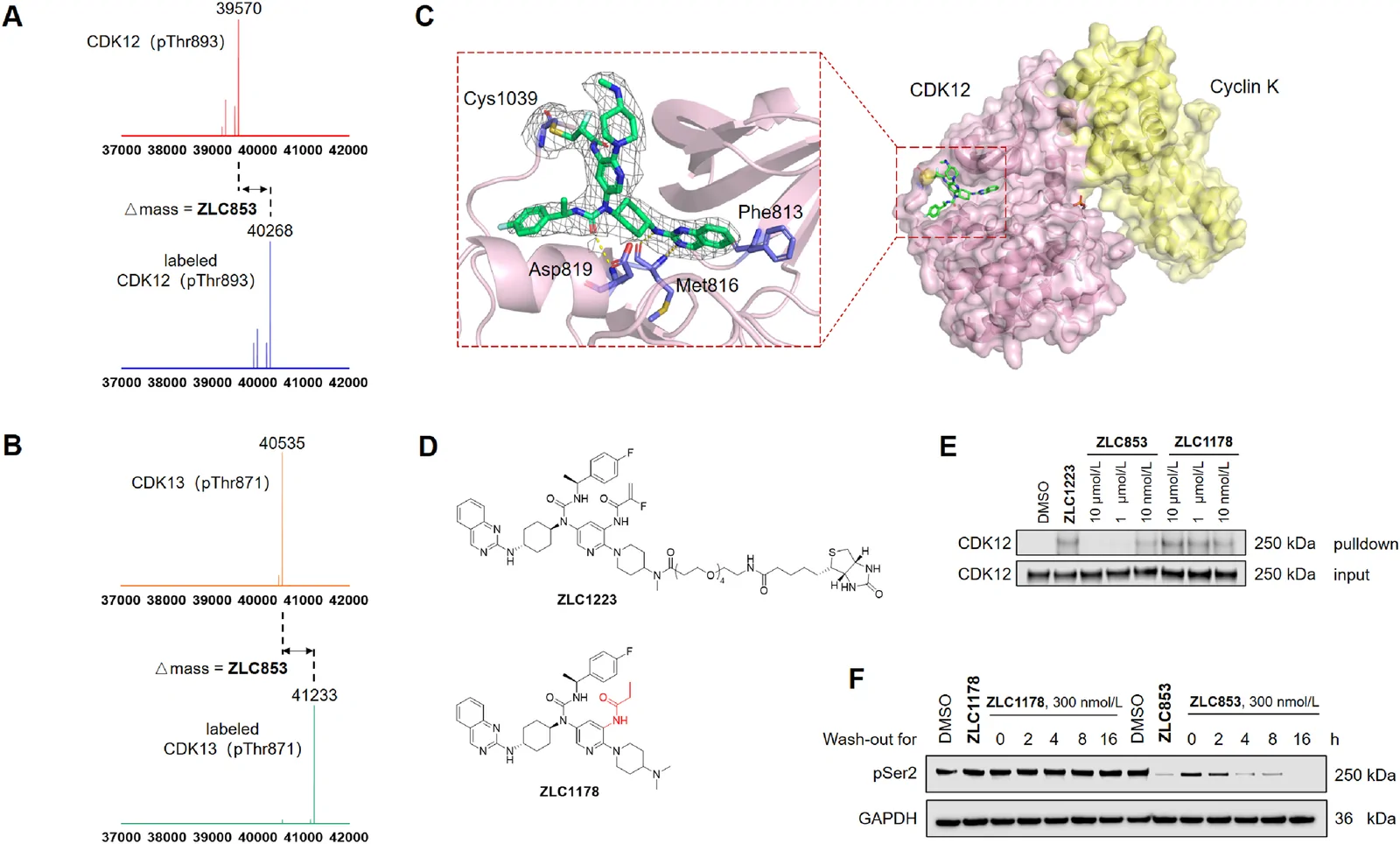
Covalent binding mode validation of **ZLC853** (A, B) Mass spectral peaks of CDK12 (A) and CDK13 (B). CDK12/Cyclin K and CDK13/Cyclin K protein complexes were treated with DMSO (top) or a 5-fold molar excess of **ZLC853** (bottom) overnight at room temperature (C) Co-crystal structure of the CDK12/Cyclin K complex bound to **ZLC853** (PDB: 9X26). **ZLC853** is shown as green sticks, and key CDK12 residues are shown as purple sticks. Hydrogen bonds to key amino acids are indicated by yellow dashed lines (D) Chemical structure of **ZLC1223** (a biotinylated analog of **ZLC853**) and **ZLC1178** (a reversible counterpart of **ZLC853**) (E) Results of the pull-down assay. VCaP cells were pretreated with **ZLC853** or the control compound for 2 h, followed by washout and incubation with **ZLC1223** for 4 h (F) Washout experiment in VCaP cells treated with 300 nmol/L **ZLC853** or **ZLC1178**.

### 2.4 **ZLC853** exhibits high kinase selectivity for CDK12 and CDK13

To evaluate its target specificity, **ZLC853** was profiled against a panel of 416 kinases at a concentration of 1 μmol/L (Fig. 5 and Supporting Information Table S1). The results demonstrate that **ZLC853** is a selective inhibitor of CDK12 and CDK13, with only five additional kinases exhibiting inhibition greater than 90% (Table S2). Notably, within the CDK family, **ZLC853** showed >90% inhibition and a similar IC_50_ value only against CDK7, an actively exploring target for drug discovery, aside from CDK12/13. To confirm this effect, we conducted the pulldown assay in cells using **ZLC1223**. The results showed that a 4-h incubation of VCaP cells with 3 μmol/L **ZLC1223** revealed robust enrichment of CDK12/13 and their partner CCNK, whereas CDK7 was not appreciably captured, indicating that **ZLC853** does not engage CDK7 covalently in cells (Supporting Information Fig. S3). For other CDK family members, **ZLC853** showed over 16- and 36-fold selectivity for CDK12/13, respectively, based on the IC_50_ values (Fig. 5B).

**Figure 5:**
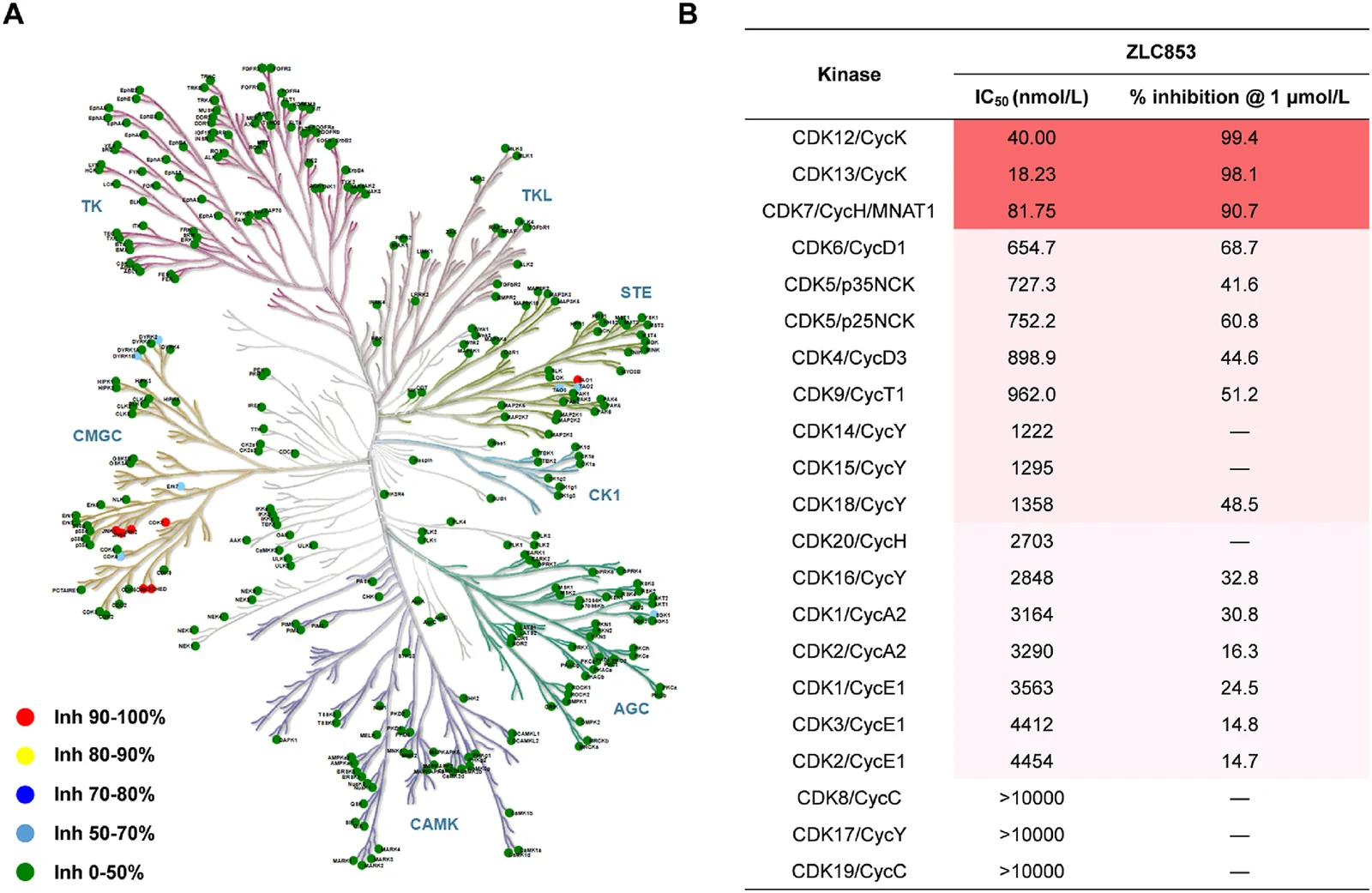
Kinase selectivity of **ZLC853** (A) KinomeScan kinase selectivity profiles for **ZLC853**. Compound **ZLC853** was profiled at a concentration of 1.0 μmol/L against a diverse panel of 416 kinases by ICE (B) IC_50_ values and inhibition percentage of compound **ZLC853** against CDK family members. The data are the means from two independent experiments.

### 2.5 **ZLC853** demonstrates favorable PK profiles

Due to its compelling target inhibition and selectivity profiles, we assessed the liver microsomal stability of **ZLC853** across different species. The results revealed that **ZLC853** showed slow metabolism in human and rat liver microsomes, with the *t*_1/2_ being 2–5 times longer than that in mouse, dog or monkey (Fig. 6A). Metabolite identification studies also elucidated the metabolic pathways of **ZLC853**, and the *N*-demethylation product (M13) was identified as the predominant metabolite in liver microsomes across all species of mouse, rat, dog, monkey and human liver microsomes (Supporting Information Fig. S4). Cytochrome P450 (CYP450) Phenotyping Screening assay further identified CYP3A as the dominant metabolic enzyme (Supporting Information Table S3). **ZLC853** was then advanced into rat PK studies (Table 1). It demonstrated significantly improved PK parameters compared to YJZ5118, including a longer half-life (*T*_1/2_ = 6.44 h *VS* 1.42 h), a higher maximum concentration (*C*_max_ = 409 ng/mL *VS* 174 ng/mL), greater oral exposure (AUC_(0–t)_ = 5248 h‧ng/mL *VS* 526 h‧ng/mL), and superior oral bioavailability (*F* = 66.2% *vs* 13.8%). The *in vitro* ADME profile of **ZLC853** also demonstrated favorable physicochemical properties, including a Caco-2 permeability of 4.83 × 10^−6^ cm/s, an efflux ratio of 1.87, and a free fraction of rat plasma protein binding of 5.6% (Fig. 6B).

**Figure 6:**
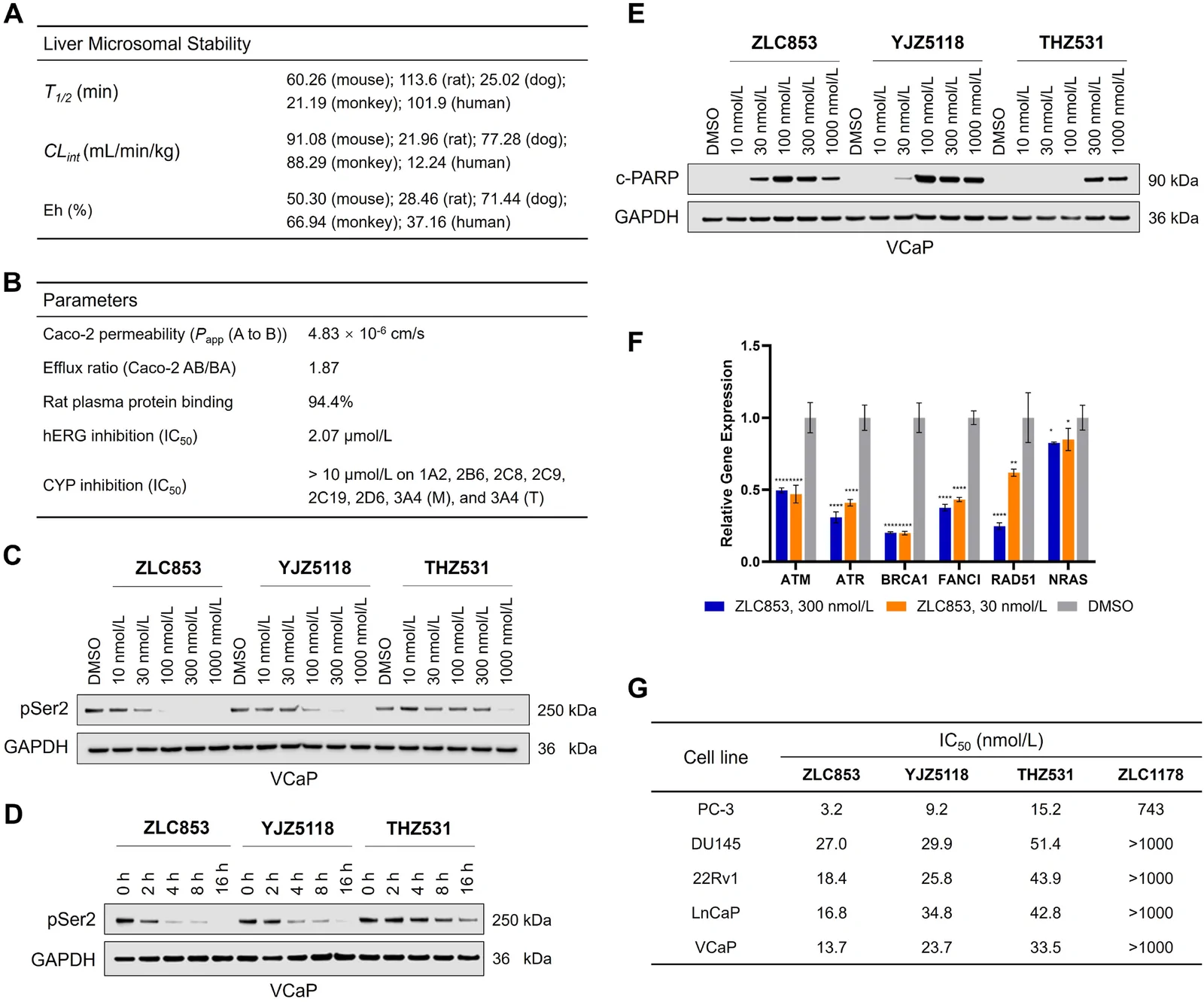
**ZLC853** inhibits Ser 2 phosphorylation of RNA polymerase II, suppresses transcription of DDR genes, and induces apoptosis (A) Liver microsomal stability results of **ZLC853** across five species (B) Caco-2 permeability, plasma protein binding, hERG and CYP inhibition assay results of **ZLC853** (C) Immunoblots showing phosphorylation of Ser2 on the RNA polymerase II CTD in VCaP cells treated with **ZLC853**, YJZ5118 or THZ531 at the indicated concentrations for 4 h (D) Immunoblots of pSer 2-RNA polymerase II CTD in VCaP cells treated with 100 nmol/L **ZLC853**, YJZ5118 or THZ531 for the indicated time points (E) Immunoblots of cleaved PARP (c-PARP) in VCaP cells treated with **ZLC853**, YJZ5118 or THZ531 at the indicated concentrations for 24 h. c-PARP is the proteolytic fragment generated by caspase-3/7-mediated cleavage of PARP-1, serving as a definitive biomarker for indicating the activation of apoptotic cascade. GAPDH served as a loading control for panels C–E (F) Quantitative PCR (qRCR) analysis of the indicated DDR gene expression in VCaP cells treated with **ZLC853** for 6 h at the indicated concentrations. Data are represented as mean ± SD from three biological replicates. ∗*P* ≤ 0.05, ∗∗*P* ≤ 0.01, ∗∗∗∗*P* ≤ 0.0001 by t-test (G) Inhibition of cell growth by **ZLC853.**

### 2.6 **ZLC853** inhibits Ser 2 phosphorylation of RNA polymerase II, suppresses transcription of DDR genes, induces apoptosis, and reduces prostate cancer cell proliferation

In VCaP cells, **ZLC853** potently inhibited phosphorylation at Ser 2 of the RNA pol II CTD in a dose- and time-dependent manner, with nearly complete loss of phosphorylation observed after treatment with 100 nmol/L for 4 h (Fig. 6C and D). Notably, **ZLC853** induced apoptosis after 24 h of treatment at 30 nmol/L, an effect comparable to YJZ5118 and superior to THZ531 (Fig. 6E). To evaluate whether CDK12/13 functional inhibition mediates downregulation of DDR gene expression, quantitative polymerase chain reaction (qPCR) was performed in cells treated with varying concentrations of **ZLC853**. Treatment of VCaP cells with **ZLC853** for 6 h significantly reduced the expression of multiple DDR genes—including Ataxia Telangiectasia Mutated (*ATM*), Ataxia Telangiectasia and Rad3 Related (*ATR*), Breast Cancer Susceptibility Gene Type 1 (*BRCA1*), Fanconi Anemia Complementation Group 1 (*FANC1*), and *RAD51,* at concentrations as low as 30 nmol/L. In contrast, expression of the cancer-related gene *NRAS* remained unaffected (Fig. 6F). We also evaluated the preliminary *in vitro* antitumor efficacy of **ZLC853** by assessing its ability to inhibit viability across a panel of prostate cancer cell lines. The results indicated that multiple prostate cancer cell lines were highly sensitive to **ZLC853**, exhibiting IC_50_ values below 50 nmol/L. In contrast, the reversible inhibitor **ZLC1178** showed markedly reduced antiproliferative activity (Fig. 6G). These results demonstrate the potent *in vitro* antitumor activity of **ZLC853**.

### 2.7 **ZLC853** demonstrates promising safety profiles in *vitro* and *in vivo* studies

*In vitro* safety profiling showed no obvious inhibition of CYP450 enzymes across all tested isoforms (IC_50_ > 10.0 μmol/L), and moderate inhibition on hERG (IC_50_ = 2.07 μmol/L) (Fig. 6F). To assess the *in vivo* efficacy and safety of **ZLC853**, we conducted comprehensive toxicity and maximum tolerance dose (MTD) studies by administering the compound to male and female CD-1 mice for three weeks. The MTD was determined to be 20 mg/kg, based on a body weight loss exceeding 20% from baseline (Supporting Information Fig. S5). At the dose of 10 mg/kg, mice appeared healthy, with mild leukocytosis and transaminase elevations constituting the only laboratory abnormalities, and no evidence of nephrotoxicity (Supporting Information Fig. S5 and S6).

### 2.8 **ZLC853** inhibits prostate cancer growth in mouse xenograft models

We then performed *in vivo* efficacy testing of **ZLC853** in a VCaP CRPC mouse model (Fig. 7A). Compared to vehicle controls, mice treated with **ZLC853** showed a significant reduction in tumor growth **(**Fig. 7B), with tumor growth inhibition of 76.6%, 89.1% and 99.5% at doses of 2, 5 and 10 mg/kg, respectively. Animals displayed no significant body weight changes (Fig. 7C). Tumor tissues were harvested at the experiment endpoint (Day 26) and subjected to histological analysis, qPCR and Western blotting. H&E staining of xenograft sections demonstrated **ZLC853** induced hyalinization and degeneration in neoplastic cell clusters. Immunohistochemistry for Ki67 showed that **ZLC853** reduced tumor cell proliferation in a dose-dependent manner (Fig. 7D and E). Additionally, qPCR demonstrated downregulated expression of multiple DDR genes—including *ATM*, *FANC1*, *RAD51* and *BRCA1*—in tumors from **ZLC853**-treated mice (Fig. 7F). The *in vivo* inhibition of CDK12/13 function by compound **ZLC853** was further supported by the observation that phosphorylation of Ser 2 on RNA Pol II CTD declined at the 5 and 10 mg/kg doses (Fig. 7G).

**Figure 7:**
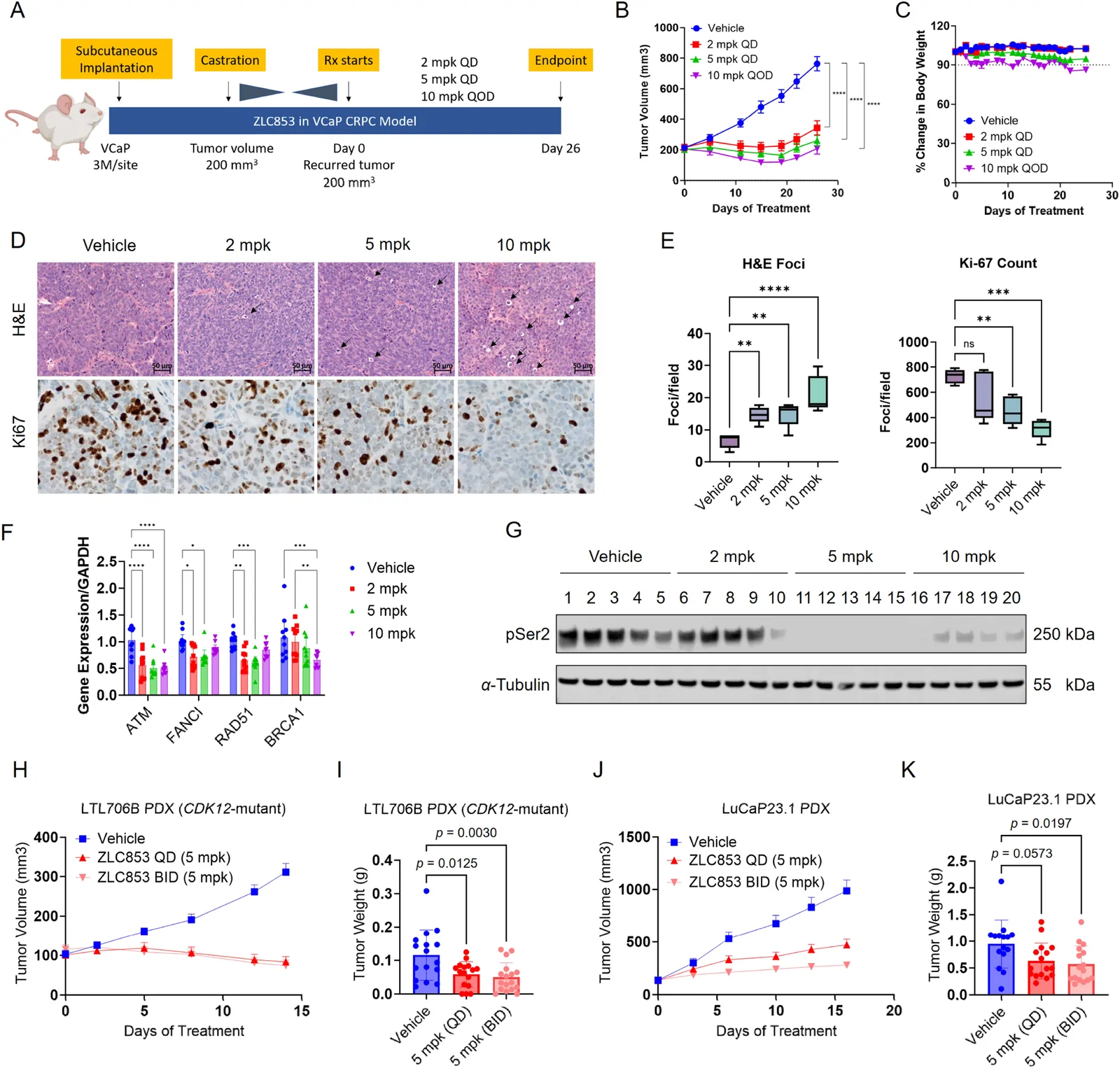
*In vivo* efficacy investigation of **ZLC853** (A) Study design for pharmacodynamic testing of **ZLC853** in the VCaP CRPC mouse model (B) Tumor volume measurements in mice treated with **ZLC853** (*p.o.*, q.d. or q.o.d.). Data are presented as mean ± SEM; *n* = 14–16 (C) Percentage of mouse body weight throughout the treatment period. Data are presented as mean ± SEM; *n* = 10 (D) Representative H&E and Ki67 staining for tumors after 26 days of treatment (E) H&E and Ki67 histology scores analyzed and plotted by GraphPad Prism 10.0 (F) Expression of indicated genes (real-time quantitative PCR assay) in tumors after the treatment of compound **ZLC853** for 26 days in a VCaP CRPC model. Data are presented as mean values ± SD of triplicate points. ∗*P* ≤ 0.05, ∗∗*P* ≤ 0.01, ∗∗∗*P* ≤ 0.001, ∗∗∗∗*P* ≤ 0.0001 by *t*-test (G) Immunoblots of pSer 2 of RNA polymerase II CTD from tumor tissue lysate of VCaP CRPC model after 26 days of treatment with **ZLC853**. *α*-Tubulin loading control (H–K) Tumor volume and weight measurements in PDX models treated with 5 mg/kg (q.d. *vs* b. i.d.) of **ZLC853**. Data are presented as mean ± SEM; *n* = 12–16.

We further evaluated the tumor inhibitory potential of **ZLC853** in the Patient-Derived Xenograft (PDX) prostate cancer models LTL706B (which harbors a *CDK12* mutation) and LuCaP23.1 (which is wild type for *CDK12*). While both PDX models responded to **ZLC853** treatment, tumor growth inhibition was more pronounced in the LTL706B xenografts (Fig. 7H–K). This finding agrees with our previously published work, which demonstrated that targeting of CDK13 displayed synthetic lethal effects in *CDK12*-inactive prostate and ovarian cancer^40^^,^^41^. In summary, **ZLC853** exhibits a mechanism of action similar to previously reported CDK12/13 inhibitors or degraders but offers markedly improved oral bioavailability, underscoring its potential as a superior therapeutic candidate.

## 3 Discussion and conclusion

Previous work by our group and others has established that inactivating mutations of CDK12 define distinct molecular subtypes in advanced prostate cancer and ovarian cancer, and uncovered a paralog synthetic lethal relationship between CDK12 and CDK13^40^, ^41^, ^42^. In line with this, dual targeting of CDK12/13—either by CRISPR-mediated gene editing or through degradation strategies—produced more robust antitumor responses than selective inhibition of either kinase alone^41^. Importantly, combined CDK12/13 targeting did not elicit obvious toxicity in animal models. Moreover, we identified STING activation as the key mechanism driving T-cell infiltration in CDK12-mutant cancers, suggesting that CDK12/13 inhibition activates STING signaling and could potentially overcome resistance to immunotherapies, particularly immune checkpoint blockade^43^. Collectively, these findings highlight CDK12 and CDK13 as promising targets for cancer therapy.

Substantial efforts have been directed toward developing CDK12/13 modulators, with only CT7439 (Carrick Therapeutics), a cyclin K-targeting molecular glue degrader, progressing into clinical trials. Several orally bioavailable CDK12/13 PROTAC degraders were also developed with promising potential. Covalent inhibitors represent a promising class of kinase-targeting agents, offering prolonged target engagement and enhanced cellular activity through irreversible binding^44^. The presence of unique cysteine residues near the ATP-binding pockets of CDK12 (Cys 1039) and CDK13 (Cys 1017) provides an opportunity to achieve high selectivity *via* covalent targeting. However, despite the development of several potent covalent CDK12/13 inhibitors, advancement has been hampered by limited selectivity or unfavorable PK properties.

Here, we report the discovery of **ZLC853**, a potent, selective, and orally bioavailable covalent inhibitor of CDK12/13. This compound inhibited CDK12 and CDK13 with IC_50_ values of 40.0 and 18.2 nmol/L, respectively, and exhibited high selectivity across the CDK family. **ZLC853** demonstrated cytotoxicity at nanomolar concentrations across various prostate cancer cells. Mechanistically, **ZLC853** rapidly reduced the phosphorylation of RNA pol II CTD Ser 2 and downregulated DDR genes. The compound achieved significantly improved pharmacokinetic properties with an oral bioavailability of 66.2% in rats. *In vivo*, **ZLC853** demonstrated antitumor efficacy in multiple CRPC xenograft models following oral administration. Notably, it achieved more pronounced tumor suppression in a CDK12-mutant prostate cancer PDX model (LTL706B) compared to a CDK12 wild-type model (LuCaP23.1). In summary, our findings establish **ZLC853** as a candidate for clinical CDK12/13-targeted cancer therapy.

## 4 Experimental

### 4.1 General methods for chemistry

All reagents and solvents in chemical synthesis were commercially available and used without further purification, unless indicated otherwise. All reactions were monitored by thin-layer chromatography (TLC) and visualized by UV light visualization (254 and 365 nmol/L). Compounds were purified by column chromatography on silica gel (200–300 mesh). The ^1^H and ^13^C NMOL/LR spectra were recorded on an Agilent DD2 500 spectrometer (Agilent Technologies Inc., Santa Clara, USA) or a Bruker AVANCE 600 spectrometer (Bruker Company, Karlsruhe, Germany) and referenced with respect to appropriate internal standards or residual solvent peaks (CDCl_3_ = 7.26 ppm, DMSO-*d*_6_ = 2.50 ppm). The following abbreviations were used in reporting spectra: s (singlet), d (doublet), t (triplet), q (quartet), and m (multiplet). The spectra of high-resolution mass (HRMS) were monitored by Bruker MaXis 4G TOF Mass Spectrometer (Bruker Company, Karlsruhe, Germany) with an ESI source. The purities of all final compounds were monitored by high-performance liquid chromatography (HPLC) analysis with the Agilent 1200 system. HPLC condition: Triart C18 reversed-phase column, 5 μm, 4.6 mm × 250 mm, and flow rate 1.0 mL/min, starting with a 15 min gradient from 0.1% TFA in water and acetonitrile 9:1 mixture to 0.1% TFA in acetonitrile, then ending with 0.1% TFA in acetonitrile for 5 min. The purity of all final compounds was confirmed to be >95% by HPLC analysis with the Agilent 1260 system.

General procedure for the synthesis of **3a** and **3b**. To a stirred solution of 4-bromo-1-fluoro-2-nitrobenzene (4.5 g, 20.5 mmol) in acetonitrile (10 mL) were added *N*,*N*-dimethylpiperidin-4-amine (2.63 g, 20.5 mmol) and Cs_2_CO_3_ (8.0 g, 24.6 mmol). The mixture was heated at 60 °C overnight under stirring. After cooling to rt, the reaction solution was filtered. The solvent was evaporated under reduced pressure and purified using column chromatography to afford target **3a** (6.36 g, 95 %). Compound **3b** was prepared by a procedure similar to that used for compound **3a**.

*1-(4-Bromo-2-nitrophenyl)-N,N-dimethylpiperidin-4-amine (****3a****).* ^1^H NMOL/LR (500 MHz, DMSO-*d*_6_) *δ* 7.98 (d, *J* = 2.5 Hz, 1H), 7.69 (dd, *J* = 8.9, 2.5 Hz, 1H), 7.23 (d, *J* = 8.9 Hz, 1H), 3.19 – 3.16 (m, 1H), 2.81 – 2.75 (m, 2H), 2.22 – 2.17 (m, 7H), 1.79 – 1.76 (m, 2H), 1.49 – 1.40 (m, 2H). MS (ESI) for C_13_H_18_BrN_3_O_2_ [M+H]^+^, calcd: 328.1, found: 328.2.

*1-(5-Bromo-3-nitropyridin-*2-yl*)-N,N-dimethylpiperidin-4-amine (****3b****).* ^1^H NMOL/LR (500 MHz, DMSO-*d*_6_) *δ* 8.48 (d, *J* = 2.3 Hz, 1H), 8.42 (d, *J* = 2.3 Hz, 1H), 3.71 (dd, *J* = 12.8, 4.1 Hz, 2H), 3.01 (dd, *J* = 13.5, 11.6, 2.7 Hz, 2H), 2.34 (tt, *J* = 10.7, 3.8 Hz, 1H), 2.17 (s, 5H), 1.80 (dd, *J* = 13.3, 3.5 Hz, 2H), 1.41 (qd, *J* = 11.7, 4.0 Hz, 2H).

General procedure for the synthesis of **4a** and **4b**. To a solution of **3a** (1.6 g, 4.9 mmol) in anhydrous toluene (22 mL) were added *trans*-*N*-Boc-1,4-cyclohexanedimine (3.2 g, 14.7 mmol), cuprous iodide (100 mg, 0.1 mmol), K_3_PO_4_ (2.1 g, 9.8 mmol) and d-proline (57.5 mg, 0.5 mmol). The resulting suspension was then evacuated and backfilled with argon (3 cycles) and heated at 100 °C overnight under stirring. After cooling to rt, the reaction solution was filtered. The solvent was evaporated under reduced pressure and purified using column chromatography to afford target **4a**. Compound **4b** was prepared by a procedure similar to that used for compound **4a** (1.6 g, 71%).

*tert-Butyl ((1R,4R)-4-((4-(4-(dimethylamino)piperidin-*1-yl*)-3-nitrophenyl)amino)cyclohexyl)carbamate (****4a****).* ^1^H NMOL/LR (500 MHz, DMSO-*d*_6_) *δ* 7.16 (d, *J* = 8.9 Hz, 1H), 6.83 (d, *J* = 2.7 Hz, 1H), 6.80 – 6.75 (m, 2H), 5.78 (d, *J* = 8.1 Hz, 1H), 3.24 – 3.17 (m, 1H), 3.11 – 3.06 (m, 1H), 2.97 (d, *J* = 11.4 Hz, 2H), 2.64 (t, *J* = 10.7 Hz, 2H), 2.18 (m, 7H), 1.93 (d, *J* = 12.0 Hz, 2H), 1.76 (m, 4H), 1.42 (m, 2H), 1.38 (s, 9H), 1.31 – 1.21 (m, 2H), 1.13 (m, 2H). MS (ESI) for C_24_H_39_N_5_O_4_ [M+H]^+^, calcd: 462.3, found: 462.4.

*tert-Butyl ((1R,4R)-4-((6-(4-(dimethylamino)piperidin-*1-yl*)-5-nitropyridin-*3-yl*)amino)cyclohexyl)carbamate (****4b****).* ^1^H NMOL/LR (500 MHz, DMSO-*d*_6_) *δ* 7.94 (d, *J* = 2.6 Hz, 1H), 7.39 (d, *J* = 2.7 Hz, 1H), 6.78 (d, *J* = 7.8 Hz, 1H), 5.73 (d, *J* = 8.0 Hz, 1H), 3.35 (s, 2H), 3.21 – 3.14 (d, *J* = 8.1 Hz, 1H), 3.12 – 3.10 (d, *J* = 9.8 Hz, 1H), 2.79 – 2.71 (m, 2H), 2.20 – 2.17 (m, 7H), 1.92 (d, *J* = 12.5 Hz, 2H), 1.76 (d, *J* = 12.4 Hz, 4H), 1.45 – 1.39 (m, 2H), 1.36 (s, 9H), 1.27 – 1.22 (m, 1H), 1.16 – 1.08 (m, 2H).

General procedure for the synthesis of **5a** and **5b**. To a solution of compound **4a** (1.12 g, 3.1 mmol) in anhydrous CH_2_Cl_2_ (2 mL) was added TFA (1 mL). The mixture was stirred for 1 h. Then the reaction was evaporated under reduced pressure. The residue was used directly in the next step without further purification. To a solution of the residue in DMF (10 mL) was added Cs_2_CO_3_ (3 g, 9.2 mmol) and 2-chloroquinazoline (512 mg, 3.1 mmol). The resulting solution was heated at 60 °C for 6 h under stirring. Upon cooling, the reaction solution was filtered. The solvent was evaporated under reduced pressure and purified using column chromatography to afford target **5a**. Compound **5b** was prepared by a procedure similar to that used for compound **5a** (924 mg, 61%).

*(1R,4R)-N1-(4-(4-(Dimethylamino)piperidin-*1-yl*)-3-nitrophenyl)-N4-(quinazolin-*2-yl*)cyclohexane-1,4-diamine (****5a****).* ^1^H NMOL/LR (500 MHz, DMSO-*d*_6_) *δ* 9.09 (s, 1H), 7.76 (d, *J* = 7.9 Hz, 1H), 7.68 – 7.65 (m, 1H), 7.45 (d, *J* = 7.2 Hz, 1H), 7.34 (d, *J* = 7.6 Hz, 1H), 7.21 – 7.17 (m, 2H), 6.88 (d, *J* = 2.5 Hz, 1H), 6.81 (dd, *J* = 8.9, 2.5 Hz, 1H), 5.86 (d, *J* = 8.0 Hz, 1H), 3.90 – 3.82 (m, 1H), 3.23 – 3.14 (m, 1H), 3.01 – 2.98 (m, 2H), 2.68 – 2.64 (m, 2H), 2.38 – 2.22 (m, 7H), 2.02 – 2.00 (m, 4H), 1.80 (d, *J* = 12.0 Hz, 2H), 1.49 – 1.41 (m, 4H), 1.28 – 1.24 (m, 2H). MS (ESI) for C_27_H_35_N_7_O_2_ [M+H]^+^, calcd: 490.3, found: 490.4.

*(1R,4R)-N1-(6-(4-(Dimethylamino)piperidin-*1-yl*)-5-nitropyridin-*3-yl*)-N4-(quinazolin-*2-yl*)cyclohexane-1,4-diamine (****5b****).* ^1^H NMOL/LR (500 MHz, DMSO-*d*_6_) *δ* 9.09 (s, 1H), 8.01 (d, *J* = 2.7 Hz, 1H), 7.77 (d, *J* = 8.0 Hz, 1H), 7.69 – 7.64 (m, 1H), 7.47 (d, *J* = 2.7 Hz, 1H), 7.44 (d, *J* = 8.9 Hz, 1H), 7.35 (d, *J* = 8.0 Hz, 1H), 7.20 (t, *J* = 7.4 Hz, 1H), 5.90 (d, *J* = 8.1 Hz, 1H), 3.95 – 3.87 (m, 1H), 3.41 (d, *J* = 12.7 Hz, 2H), 3.26 – 3.23 (m, 2H), 2.81 (t, *J* = 12.1 Hz, 2H), 2.55 – 2.51 (m, 4H), 2.02 (d, *J* = 11.8 Hz, 4H), 1.93 (d, *J* = 12.3 Hz, 2H), 1.56 (d, *J* = 11.9 Hz, 2H), 1.45 (q, *J* = 12.2 Hz, 2H), 1.28 (q, *J* = 12.7, 12.3 Hz, 2H).

General procedure for the synthesis of **6a**–**c**. To a solution of **5a** (264 mg, 0.54 mmol) and DIPEA (0.28 mL, 1.62 mmol) in DMF (2 mL) was added benzyl isocyanate (215 mg, 1.62 mmol) at room temperature. The mixture was stirred at 90 °C for 16 h. The solvent was removed under reduced pressure and purified by column chromatography to afford target **6a** (225 mg, 67 %)**.** Compounds **6b**–**c** were prepared by a procedure similar to that used for compound **6a**.

*3-Benzyl-1-(4-(4-(dimethylamino)piperidin-*1-yl*)-3-nitrophenyl)-1-((1R,4R)-4-(quinazolin-2-ylamino)cyclohexyl)urea (****6a****).* ^1^H NMOL/LR (500 MHz, DMSO-*d*_6_) *δ* 9.05 (s, 1H), 7.74 (d, *J* = 8.1 Hz, 1H), 7.65 – 7.59 (m, 2H), 7.43 – 7.36 (m, 2H), 7.34 – 7.31 (m, 1H), 7.29 – 7.24 (m, 3H), 7.18 (m, 4H), 6.26 – 6.23 (m, 1H), 4.29 – 4.24 (m, 1H), 4.16 (d, *J* = 6.2 Hz, 2H), 3.64 – 3.57 (m, 1H), 3.28 (m, 2H), 2.89 – 2.85 (m, 2H), 2.36 – 2.19 (m, 7H), 2.00 – 1.92 (m, 2H), 1.88 – 1.81 (m, 4H), 1.55 – 1.48 (m, 2H), 1.45 – 1.38 (m, 2H), 1.17 – 1.10 (m, 2H). MS (ESI) for C_35_H_42_N_8_O_3_ [M+H]^+^, calcd: 623.3, found: 623.7.

*1-(6-(4-(Dimethylamino)piperidin-*1-yl*)-5-nitropyridin-*3-yl*)-3-((R)-1-phenylethyl)-1-((1R,4R)-4-(quinazolin-2-ylamino)cyclohexyl)urea (****6b****).* ^1^H NMOL/LR (500 MHz, DMSO-*d*_6_) *δ* 9.04 (s, 1H), 8.19 (d, *J* = 2.4 Hz, 1H), 7.95 (d, *J* = 2.4 Hz, 1H), 7.74 (d, *J* = 8.0 Hz, 1H), 7.63 (t, *J* = 7.7 Hz, 1H), 7.39 (d, *J* = 8.5 Hz, 1H), 7.28 (t, *J* = 7.5 Hz, 2H), 7.25 – 7.21 (m, 2H), 7.18 (t, *J* = 7.4 Hz, 2H), 6.27 (d, *J* = 8.2 Hz, 1H), 4.89 (q, *J* = 7.3 Hz, 1H), 4.23 (t, *J* = 12.4 Hz, 1H), 3.82 (d, *J* = 13.0 Hz, 2H), 3.61 (s, 1H), 3.06 (t, *J* = 12.3 Hz, 2H), 2.41 – 2.33 (m, 1H), 2.21 (s, 6H), 2.05 – 1.90 (m, 2H), 1.91 – 1.71 (m, 4H), 1.53 – 1.46 (m, 2H), 1.42 – 1.35 (m, 2H), 1.28 (d, *J* = 7.1 Hz, 3H), 1.16 – 1.04 (m, 2H)

*1-(6-(4-(Dimethylamino)piperidin-*1-yl*)-5-nitropyridin-*3-yl*)-3-((S)-1-phenylethyl)-1-((1R,4S)-4-(quinazolin-2-ylamino)cyclohexyl)urea (****6c****).* ^1^H NMOL/LR (500 MHz, DMSO-*d*_6_) *δ* 9.04 (s, 1H), 8.19 (s, 1H), 7.95 (s, 1H), 7.74 (d, *J* = 7.8 Hz, 1H), 7.63 (t, *J* = 7.6 Hz, 1H), 7.39 (d, *J* = 8.5 Hz, 1H), 7.27 (d, *J* = 7.8 Hz, 2H), 7.23 (d, *J* = 7.3 Hz, 3H), 7.18 (d, *J* = 8.0 Hz, 2H), 6.31 – 6.24 (m, 1H), 4.89 (t, *J* = 8.0 Hz, 1H), 4.24 (d, *J* = 13.0 Hz, 1H), 3.82 (d, *J* = 13.0 Hz, 2H), 3.62 (s, 1H), 3.05 (t, *J* = 12.8 Hz, 2H), 2.39 (s, 1H), 2.252 (m, 6H), 2.01 – 1.95 (m, 2H), 1.87 – 1.72 (m, 4H), 1.50 (d, *J* = 12.2 Hz, 2H), 1.42 – 1.38 (m, *J* = 12.3 Hz, 2H), 1.29 – 1.26 (m, 3H), 1.16 – 1.08 (m, 2H).

*1-(6-(4-(Dimethylamino)piperidin-*1-yl*)-5-nitropyridin-*3-yl*)-3-((S)-1-(4-fluorophenyl)ethyl)-1-((1R,4S)-4-(quinazolin-2-ylamino)cyclohexyl)urea (****6d****).* To a stirred solution of compound *(S)*-1-(4-fluorophenyl) ethanamine (540 mg, 3.88 mmol) in anhydrous THF (8 mL) were added Et_3_N (1.1 mL, 7.76 mmol) and the THF solution (8 mL) of 4-nitrophenyl chloroformate (780 mg, 3.88 mmol). The resulting solution was stirred for 2 h at room temperature. After the reaction was completed as indicated by TLC, the mixture was filtered. The solvent was evaporated under reduced pressure, and the residue was used directly in the next step without further purification. To a solution of compound **5b** (1.08 g, 2.2 mmol) in DMF (10 mL) was added the residue (3.3 g), DIPEA (1.15 mL) and DMAP (27 mg). The mixture was stirred at 90 °C for 16 h. After the reaction was completed as indicated by TLC, the mixture was filtered. The solvent was removed under reduced pressure and purified by column chromatography to afford target **6d** (949 mg, 66 %). ^1^H NMOL/LR (500 MHz, DMSO-*d*_6_) *δ* 9.04 (s, 1H), 8.18 (d, *J* = 2.4 Hz, 1H), 7.94 (d, *J* = 2.4 Hz, 1H), 7.74 (dd, *J* = 8.0, 1.6 Hz, 1H), 7.66 – 7.60 (m, 1H), 7.39 (d, *J* = 8.5 Hz, 1H), 7.26 (td, *J* = 10.5, 9.6, 6.6 Hz, 3H), 7.17 (dd, *J* = 8.0, 6.8, 1.1 Hz, 1H), 7.13 – 7.07 (m, 2H), 6.28 (d, *J* = 8.2 Hz, 1H), 4.88 (*P*, *J* = 7.3 Hz, 1H), 4.27 – 4.18 (m, 1H), 3.82 (d, *J* = 13.0 Hz, 2H), 3.62 (s, 1H), 3.10 – 3.01 (m, 2H), 2.37 (d, *J* = 10.9 Hz, 1H), 2.21 (s, 6H), 1.98 (d, *J* = 10.0 Hz, 2H), 1.91 – 1.71 (m, 4H), 1.56 – 1.46 (m, 2H), 1.43 – 1.34 (m, 2H), 1.27 (d, *J* = 7.1 Hz, 3H), 1.10 (dq, *J* = 24.1, 12.4 Hz, 2H).

General procedure for the synthesis of **7a**–**d**. To a solution of **6a** (216 mg, 0.35 mmol) in DMF (1 mL) was added 4,4′-bipyridine (1 mg) and hypodiboric acid (156 mg, 1.75 mmol). The mixture was stirred for 0.5 h. And then a saturated NaHCO_3_ solution was added. The mixture was filtered and washed with H_2_O (10 mL). The filter residue was dissolved with dichloromethane/methanol (10:1, *v*/*v*) and subsequently dried over Na_2_SO_4_. The solvent was removed under reduced pressure and purified by column chromatography to afford target **7a** (135 mg, 64%). Compounds **7b**–**d** were prepared by a procedure similar to that used for compound **7a**.

*1-(3-Amino-4-(4-(dimethylamino)piperidin-*1-yl*)phenyl)-3-benzyl-1-((1R,4R)-4 (quinazolin-2-ylamino)cyclohexyl)urea (****7a****).* ^1^H NMOL/LR (500 MHz, DMSO-*d*_6_) *δ* 9.05 (s, 1H), 7.74 (d, *J* = 8.1 Hz, 1H), 7.62 (t, *J* = 7.6 Hz, 1H), 7.42 (s, 1H), 7.29 – 7.23 (m, 3H), 7.19 – 7.16 (m, 4H), 6.92 (d, *J* = 8.2 Hz, 1H), 6.56 – 6.53 (m, 1H), 6.37 (dd, *J* = 8.2, 2.1 Hz, 1H), 5.49 (m, 1H), 4.88 (s, 2H), 4.27 – 4.23 (m, 1H), 4.17 (d, *J* = 5.9 Hz, 2H), 3.65 – 3.56 (m, 1H), 3.18 (d, *J* = 11.9 Hz, 2H), 2.55 (m, 2H), 2.44 – 2.33 (m, 7H), 2.00 – 1.93 (m, 2H), 1.90 – 1.87 (m, 2H), 1.77 – 1.73 (m, 2H), 1.67 – 1.61 (m, 2H), 1.40 (m, 2H), 1.20 – 1.16 (m, 2H)

*1-(5-Amino-6-(4-(dimethylamino)piperidin-*1-yl*)pyridin-*3-yl*)-3-((R)-1-phenylethyl)-1-((1R,4R)-4-(quinazolin-2-ylamino)cyclohexyl)urea (****7b****).* ^1^H NMOL/LR (500 MHz, DMSO-*d*_6_) *δ* 9.04 (s, 1H), 7.74 (d, *J* = 8.0 Hz, 1H), 7.62 (t, *J* = 7.8 Hz, 1H), 7.41 (d, *J* = 8.5 Hz, 1H), 7.36 (d, *J* = 2.2 Hz, 1H), 7.28 (t, *J* = 7.5 Hz, 2H), 7.25 – 7.20 (m, 3H), 7.18 (td, *J* = 7.6, 2.0 Hz, 2H), 6.81 (d, *J* = 2.3 Hz, 1H), 5.47 (d, *J* = 8.1 Hz, 1H), 4.90 (s, 2H), 4.84 (*P*, *J* = 7.3 Hz, 1H), 4.21 (dd, *J* = 14.0, 10.5 Hz, 1H), 3.60 (d, *J* = 10.3 Hz, 1H), 3.51 (d, *J* = 12.1 Hz, 2H), 2.62 (t, *J* = 12.0 Hz, 2H), 2.22 – 2.16 (m, 7H), 1.94 (d, *J* = 14.5 Hz, 2H), 1.84 (d, *J* = 12.2 Hz, 2H), 1.77 – 1.67 (m, 2H), 1.65 – 1.55 (m, 2H), 1.44 – 1.36 (m, 2H), 1.26 (d, *J* = 7.3 Hz, 3H), 1.19 – 1.05 (m, 2H).

*1-(5-Amino-6-(4-(dimethylamino)piperidin-*1-yl*)pyridin-*3-yl*)-3-((S)-1-phenylethyl)-1-((1R,4S)-4-(quinazolin-2-ylamino)cyclohexyl)urea (****7c****).* ^1^H NMOL/LR (500 MHz, DMSO-*d*_6_) *δ* 9.04 (s, 1H), 7.74 (d, *J* = 7.9 Hz, 1H), 7.62 (t, *J* = 7.8 Hz, 1H), 7.41 (d, *J* = 8.5 Hz, 1H), 7.36 (d, *J* = 2.2 Hz, 1H), 7.28 (t, *J* = 7.5 Hz, 2H), 7.24 – 7.19 (m, 3H), 7.18 (td, *J* = 7.6, 2.1 Hz, 2H), 6.81 (d, *J* = 2.3 Hz, 1H), 5.47 (d, *J* = 8.1 Hz, 1H), 4.90 (s, 2H), 4.84 (p, *J* = 7.3 Hz, 1H), 4.21 (t, *J* = 12.2 Hz, 1H), 3.60 (s, 1H), 3.51 (d, *J* = 12.0 Hz, 2H), 2.62 (t, *J* = 12.2 Hz, 2H), 2.22 – 2.17 (m, 7H), 1.95 (s, 2H), 1.84 (d, *J* = 12.1 Hz, 2H), 1.73 (dd, *J* = 37.2, 12.2 Hz, 2H), 1.65 – 1.55 (m, 2H), 1.45 – 1.35 (m, 2H), 1.26 (d, *J* = 7.1 Hz, 3H), 1.18 – 1.08 (m, 2H)

*1-(5-Amino-6-(4-(dimethylamino)piperidin-*1-yl*)pyridin-*3-yl*)-3-((S)-1-(4-fluorophenyl)ethyl)-1-((1R,4S)-4-(quinazolin-2-ylamino)cyclohexyl)urea (****7d****).* ^1^H NMOL/LR (500 MHz, DMSO-*d*_6_) *δ* 9.04 (s, 1H), 7.74 (dd, *J* = 7.9, 1.5 Hz, 1H), 7.62 (t, *J* = 7.7 Hz, 1H), 7.41 (d, *J* = 8.4 Hz, 1H), 7.35 (d, *J* = 2.3 Hz, 1H), 7.26 (tt, *J* = 8.2, 4.1 Hz, 3H), 7.20 – 7.16 (m, 1H), 7.12 – 7.07 (m, 2H), 6.79 (d, *J* = 2.3 Hz, 1H), 5.57 (d, *J* = 8.1 Hz, 1H), 4.91 (s, 2H), 4.84 (*P*, *J* = 7.2 Hz, 1H), 4.20 (t, *J* = 12.1 Hz, 1H), 3.59 (s, 1H), 3.53 (d, *J* = 12.3 Hz, 2H), 2.63 (t, *J* = 12.0 Hz, 2H), 2.56 – 2.52 (m, 1H) 2.30 (s, 6H), 1.97 (d, *J* = 17.8 Hz, 2H), 1.88 (d, *J* = 12.0 Hz, 2H), 1.80 – 1.59 (m, 4H), 1.45 – 1.33 (m, 2H), 1.26 (d, *J* = 7.1 Hz, 3H), 1.17 – 1.05 (m, 2H).

General procedure for the synthesis of **1a**–**c** and **ZLC1178**. To a solution of **7a** (59 mg, 0.1 mmol) in dichloromethane (3 mL) was added DIPEA (52 μL, 0.3 mmol). The mixture was stirred at 0 °C for 15 min, and then acryloyl chloride (81 μL, 0.12 mmol) was added. The acryloyl chloride was further added to complete the reaction. Then, a saturated NaHCO_3_ solution was added and extracted with dichloromethane/methanol (10:1, *v*/*v*). The organic layer was washed with brine, dried over anhydrous Na_2_SO_4_, concentrated under reduced pressure, and purified by column chromatography to afford target **1a** (63 mg, 49%). Compounds **1b**–**c** and **ZLC1178** were prepared by a procedure similar to that used for compound **1a**.

*N-(5-(3-Benzyl-1-((1R,4R)-4-(quinazolin-2-ylamino)cyclohexyl)ureido)-2-(4-(dimethylamino)piperidin-*1-yl*)phenyl)acrylamide (****1a****).* ^1^H NMOL/LR (600 MHz, DMSO-*d*_6_) *δ* 9.04 (m, 2H), 7.96 (s, 1H), 7.73 (d, *J* = 8.8 Hz, 1H), 7.62 (t, *J* = 7.4 Hz, 1H), 7.41 (d, *J* = 7.4 Hz, 1H), 7.29 – 7.15 (m, 8H), 6.92 (dd, *J* = 8.3, 2.3 Hz, 1H), 6.75 (dd, *J* = 16.9, 10.3 Hz, 1H), 6.29 (d, *J* = 18.7 Hz, 1H), 5.81 – 5.74 (m, 2H), 4.30 – 4.26 (m, 1H), 4.18 (d, *J* = 5.9 Hz, 2H), 3.60 – 3.58 (m, 1H), 3.10 (d, *J* = 11.5 Hz, 2H), 2.67 (t, *J* = 11.1 Hz, 2H),2.38 – 2.33 (m, 7H), 1.98 – 1.96 (m, 2H), 1.90 – 1.88 (m, 2H), 1.80 – 1.74 (m, 4H), 1.45 – 1.38 (m, 2H), 1.21 – 1.15 (m, 2H). ^13^C NMOL/LR (151 MHz, DMSO) *δ* 163.20, 162.02, 158.46, 156.60, 151.18, 142.84, 141.26, 134.67, 133.19, 132.53, 132.19, 128.04, 127.84, 127.09, 126.98, 126.65, 126.24, 123.97, 121.18, 120.37, 119.50, 68.60, 61.56, 53.43, 51.03, 43.47, 41.21, 31.34, 30.39, 27.94. HRMS (ESI) for C_38_H_46_N_8_O_2_ [M+H]^+^, calcd: 647.3816, found: 647.3816. HPLC purity: 98.57 %.

*N-(2-(4-(Dimethylamino)piperidin-*1-yl*)-5-(3-((R)-1-phenylethyl)-1-((1R,4R)-4-(quinazolin-2-ylamino)cyclohexyl)ureido)pyridin-*3-yl*)acrylamide (****1b****).* ^1^H NMOL/LR (600 MHz, DMSO-*d*_6_) *δ* 9.30 (s, 1H), 9.04 (s, 1H), 7.98 (s, 1H), 7.87 (d, *J* = 2.3 Hz, 1H), 7.77 – 7.70 (m, 1H), 7.63 – 7.61 (m, 1H), 7.41 (d, *J* = 7.7 Hz, 1H), 7.31 – 7.22 (m, 5H), 7.19 – 7.15 (m, 2H), 6.76 (dd, *J* = 16.9, 10.3 Hz, 1H), 6.32 (dd, *J* = 17.0, 1.8 Hz, 1H), 5.90 (d, *J* = 7.4 Hz, 1H), 5.81 (d, *J* = 11.8 Hz, 1H), 4.86 (*P*, *J* = 7.1 Hz, 1H), 4.26 – 4.21 (m, 1H), 3.60 – 3.58 (m, 3H), 2.80 (t, *J* = 11.8 Hz, 2H), 2.69 – 2.52 (m, 1H), 2.43 (s, 6H), 1.99 – 1.92 (m, 4H), 1.83 – 1.72 (m, 4H), 1.46 – 1.37 (m, 2H), 1.28 (d, *J* = 7.1 Hz, 3H), 1.17 – 1.05 (m, 2H). ^13^C NMOL/LR (151 MHz, DMSO) *δ* 163.62, 162.02, 158.85, 156.05, 153.37, 151.60, 145.82, 144.79, 133.95, 133.49, 131.61, 128.83, 128.02, 127.84, 127.47, 126.20, 125.82, 125.08, 124.81, 121.70, 119.50, 70.85, 61.08, 54.74, 49.40, 48.51, 48.10, 41.78, 31.25, 30.41, 26.95, 22.48. HRMS (ESI) for C_38_H_47_N_9_O_2_ [M+H]^+^, calcd: 662.3925, found: 662.3923. HPLC purity: 97.59 % [*α*]_D_^25^ = −64.4 (c = 1.5 mg/mL, CHCl_3_).

*N-(2-(4-(Dimethylamino)piperidin-*1-yl*)-5-(3-((S)-1-phenylethyl)-1-((1R,4S)-4-(quinazolin-2-ylamino)cyclohexyl)ureido)pyridin-*3-yl*)acrylamide (****1c****).* ^1^H NMOL/LR (600 MHz, DMSO-*d*_6_) *δ* 9.30 (s, 1H), 9.04 (s, 1H), 7.98 (s, 1H), 7.87 (s, 1H), 7.73 (d, *J* = 7.9 Hz, 1H), 7.62 (t, *J* = 7.2 Hz, 1H), 7.41 (d, *J* = 6.8 Hz, 1H), 7.31 – 7.22 (m, 5H), 7.17 (t, *J* = 7.1 Hz, 2H), 6.76 (dd, *J* = 16.8, 10.3 Hz, 1H), 6.33 – 6.30 (m, 1H), 5.90 (d, *J* = 6.9 Hz, 1H), 5.81 (d, *J* = 10.5 Hz, 1H), 4.86 (*P*, *J* = 7.1 Hz, 1H), 4.25 – 4.21 (m, 1H), 3.59 (m, 3H), 2.80 (t, *J* = 12.0 Hz, 2H), 2.72 – 2.57 (m, 1H), 2.43 (s, 6H), 1.99 – 1.91 (m, 4H), 1.85 – 1.70 (m, 4H), 1.47 – 1.36 (m, 2H), 1.27 (d, *J* = 7.0 Hz, 3H), 1.17 – 1.05 (m, 2H). ^13^C NMOL/LR (151 MHz, DMSO) *δ* 164.11, 162.02, 158.86, 156.06, 153.37, 152.26, 145.82, 144.79, 133.96, 133.50, 131.61, 128.83, 128.03, 127.84, 127.48, 126.20, 125.82, 125.08, 124.81, 121.71, 119.50, 69.79, 61.98, 59.76, 53.57, 49.40, 48.51, 48.10, 41.83, 31.25, 30.41, 30.27, 26.93, 22.48. HRMS (ESI) for C_38_H_47_N_9_O_2_ [M+H]^+^, calcd: 662.3922, found: 662.3925. HPLC purity: 98.75 % [*α*]_D_^25^ = +70.2 (c = 1.5 mg/mL, CHCl_3_).

*N-(2-(4-(Dimethylamino)piperidin-1-yl)-5-(3-((S)-1-(4-fluorophenyl)ethyl)-1-((1R,4S)-4-(quinazolin-2-ylamino)cyclohexyl)ureido)pyridin-3-yl)propionamide (**ZLC1178**)*.

^1^H NMOL/LR (600 MHz, DMSO-*d*_6_) *δ* 9.04 (s, 1H), 8.90 (s, 1H), 7.87 (s, 1H), 7.82 (d, *J* = 2.4 Hz, 1H), 7.74 (dd, *J* = 7.9, 1.5 Hz, 1H), 7.62 (t, *J* = 7.8 Hz, 1H), 7.41 (d, *J* = 8.5 Hz, 1H), 7.29 – 7.25 (m, 2H), 7.24 (d, *J* = 7.8 Hz, 1H), 7.17 (dd, *J* = 8.0, 6.9, 1.1 Hz, 1H), 7.11 – 7.06 (m, 2H), 5.89 (d, *J* = 8.0 Hz, 1H), 4.84 (*P*, *J* = 7.2 Hz, 1H), 4.21 (tt, *J* = 12.0, 3.7 Hz, 1H), 3.58 (d, *J* = 8.0 Hz, 1H), 3.53 (d, *J* = 12.6 Hz, 2H), 2.75 (dd, *J* = 12.7, 9.8, 3.2 Hz, 2H), 2.49 – 2.44 (m, 2H), 2.25 – 2.18 (m, 7H), 2.01 – 1.90 (m, 2H), 1.89 – 1.82 (m, 2H), 1.79 (d, *J* = 12.3 Hz, 1H), 1.68 (dd, *J* = 25.0, 12.2 Hz, 3H), 1.46 – 1.36 (m, 2H), 1.26 (d, *J* = 7.1 Hz, 3H), 1.12 (t, *J* = 7.6 Hz, 3H), 1.11 – 1.01 (m, 2H). ^13^C NMOL/LR (151 MHz, DMSO) *δ* 172.11, 161.04, 160.59, 158.99, 155.68, 153.30, 151.03, 144.02, 141.02, 132.98, 132.04, 128.67, 127.74, 126.86, 126.79, 126.74, 124.48, 120.73, 118.51, 113.71, 113.57, 60.70, 52.58, 47.86, 47.54, 40.69, 30.30, 29.40, 29.26, 28.43, 27.07, 25.57, 21.43, 8.68. HRMS (ESI) for C_38_H_48_FN_9_O_2_ [M+H]^+^, calcd: 682.3988, found: 682.3988. HPLC purity: 98.39%.

*N-(2-(4-(Dimethylamino)piperidin-*1-yl*)-5-(3-((S)-1-(4-fluorophenyl)ethyl)-1-((1R,4S)-4-(quinazolin-2-ylamino)cyclohexyl)ureido)pyridin-*3-yl*)-2-fluoroacrylamide (****1d****).* To a solution of 2-fluoracrylic acid (200 mg, 2.22 mmol) in dichloromethane (3.1 mL) was added oxalyl chloride (127 μL, 1.55 mmol) and DMF (20 μL) at 0 °C. The mixture solution was stirred for 2 h and used directly in the next step without further purification. To a solution of **7d** (125 mg, 0.2 mmol) in dichloromethane (2 mL) was added DIPEA (0.1 mL, 0.6 mmol). The mixture was stirred at 0 °C for 15 min, and then the mixture solution (0.48 mL) was added. The solution was further added to complete the reaction. Then, a saturated NaHCO_3_ solution was added and extracted with dichloromethane/methanol (10:1, *v*/*v*). The organic layer was washed with brine, dried over anhydrous Na_2_SO_4_, concentrated under reduced pressure, and purified by column chromatography to afford target **1d** (62 mg, 46%). ^1^H NMOL/LR (600 MHz, DMSO-*d*_6_) *δ* 9.29 (s, 1H), 9.04 (s, 1H), 7.95 (dd, *J* = 17.1, 2.0 Hz, 2H), 7.73 (d, *J* = 7.8 Hz, 1H), 7.62 (t, *J* = 7.6 Hz, 1H), 7.39 (d, *J* = 8.4 Hz, 1H), 7.29 – 7.25 (m, 3H), 7.17 (t, *J* = 7.4 Hz, 1H), 7.12 – 7.06 (m, 2H), 5.99 (d, *J* = 7.5 Hz, 1H), 5.80 (dd, *J* = 48.6, 3.8 Hz, 1H), 5.53 (dd, *J* = 15.8, 3.8 Hz, 1H), 4.86 (*P*, *J* = 7.1 Hz, 1H), 4.25 – 4.21 (m, 1H), 3.56 (d, *J* = 12.1 Hz, 3H), 2.82 (t, *J* = 11.7 Hz, 2H), 2.64 – 2.53 (m, 1H), 2.38 (s, 6H), 2.05 – 1.89 (m, 4H), 1.82 (d, *J* = 12.1 Hz, 1H), 1.74 (d, *J* = 12.0 Hz, 1H), 1.63 – 1.58 (m, 2H), 1.46 – 1.37 (m, 2H), 1.27 (d, *J* = 7.1 Hz, 3H), 1.16 – 1.04 (m, 2H). ^13^C NMOL/LR (151 MHz, DMSO) *δ* 162.01, 161.59, 159.99, 158.86, 157.46, 157.25, 156.65, 155.98, 154.87, 153.49, 151.58, 145.74, 141.98, 141.96, 133.97, 132.97, 129.31, 127.85, 127.78, 127.72, 124.76, 124.23, 121.70, 119.50, 114.70, 114.56, 100.53, 100.43, 61.64, 53.62, 48.89, 48.51, 48.37, 40.88, 31.22, 30.39, 30.27, 27.78, 22.42. HRMS (ESI) for C_38_H_45_F_2_N_9_O_2_ [M+H]^+^, calcd: 698.3737, found: 698.3737. HPLC purity: 95.34 % [*α*]_D_^25^ = +83.0 (c = 2.2 mg/mL, CHCl_3_). M. p. = 172.5-173.2 °C.

*tert-Butyl((1R,4R)-4-(quinazolin-2-ylamino)cyclohexyl)carbamate (****10****).* To a solution of 2-chloroquinazoline (3.69 g, 22.4 mmol) in DMF (45 mL) was added *trans*-*N*-Boc-1,4-cyclohexanedimine (4.79 g, 22.4 mmol) and Cs_2_CO_3_ (14.6 g, 44.8 mmol). The mixture was heated at 60 °C for 6 h under stirring. After the reaction was completed as indicated by TLC, the mixture was filtered. The solvent was evaporated under reduced pressure and the residues were purified using column chromatography to afford target compound **10** (6.15 g, 80%). ^1^H NMOL/LR (500 MHz, DMSO-*d*_6_) *δ* 9.07 (s, 1H), 7.76 (d, *J* = 8.0 Hz, 1H), 7.66 (dd, *J* = 8.3, 7.0, 1.4 Hz, 1H), 7.49 – 7.40 (m, 1H), 7.23 (d, *J* = 8.1 Hz, 1H), 7.20 (t, *J* = 7.5 Hz, 1H), 6.75 (d, *J* = 8.1 Hz, 1H), 3.78 (s, 1H), 3.22 (s, 1H), 2.00 – 1.90 (m, 2H), 1.81 (d, *J* = 11.3 Hz, 2H), 1.38 (s, 9H), 1.36 – 1.24 (m, 4H).

*(1R,4R)-N1-(Quinazolin-*2-yl*)cyclohexane-1,4-diamine (****11****).* To the solution of compound **10** (6.15 g, 17.98 mmol) in CH_2_Cl_2_ (20 mL) was added TFA (10 mL). The mixture was stirred for 1 h. After the reaction was completed as indicated by TLC, the mixture was evaporated under reduced pressure. And then a saturated Na_2_CO_3_ solution was added. The mixture was filtered and washed with H_2_O (10 mL). The filter residue was dissolved with dichloromethane/methanol (10:1, *v*/*v*) and subsequently dried over Na_2_SO_4_. The solvent was removed under reduced pressure and afforded target **11** (2.13 g, 49%). ^1^H NMOL/LR (500 MHz, DMSO-*d*_6_) *δ* 9.07 (s, 1H), 7.75 (d, *J* = 8.0 Hz, 1H), 7.69 – 7.62 (m, 1H), 7.43 (d, *J* = 8.5 Hz, 1H), 7.23 – 7.15 (m, 2H), 3.78 (d, *J* = 11.1 Hz, 1H), 3.32 (s, 2H), 2.57 – 2.52 (d, *J* = 11.0 Hz, 1H), 1.92 (d, *J* = 12.5 Hz, 2H), 1.78 (d, *J* = 12.6 Hz, 2H), 1.36 – 1.27 (m, 2H), 1.17 – 1.12 (m, 2H).

*tert-Butyl(1-(5-bromo-3-nitropyridin-*2-yl*)piperidin-*4-yl*) (methyl)carbamate (****12****).* Compound **12** was prepared by a procedure similar to that used for compound **3a**. ^1^H NMOL/LR (500 MHz, DMSO-*d*_6_) *δ* 8.50 (t, *J* = 1.9 Hz, 1H), 8.44 (t, *J* = 1.9 Hz, 1H), 3.77 (d, *J* = 13.3 Hz, 2H), 3.36 – 3.32 (m, 1H), 3.05 (t, *J* = 12.7 Hz, 2H), 2.65 (d, *J* = 1.5 Hz, 3H), 1.75 – 1.56 (m, 4H), 1.40 (d, *J* = 1.5 Hz, 9H).

*tert-Butyl methyl(1-(3-nitro-5-(((1r,4r)-4-(quinazolin-2-ylamino)cyclohexyl)amino)pyridin-*2-yl*)piperidin-*4-yl*)carbamate (****13****).* To a solution of compound **11** (1.13 g, 4.67 mmol) in anhydrous toluene (20 mL) were added Pd_2_ (dba)_3_ (430 mg, 0.47 mmol), Brettphos (504 mg, 0.94 mmol), compound **12** (2 g, 4.67 mmol) and *tert*-BuONa (902 mg, 0.94 mmol). The resulting suspension was then evacuated and backfilled with argon (3 cycles) and heated at 100 °C for 12 h under stirring. After cooling, the reaction solution was filtered. The solvent was evaporated under reduced pressure and purified using column chromatography to afford target compound **13** (484 mg, 18 %). ^1^H NMOL/LR (500 MHz, DMSO-*d*_6_) *δ* 9.09 (s, 1H), 8.01 (d, *J* = 2.7 Hz, 1H), 7.77 (d, *J* = 7.9 Hz, 1H), 7.69 – 7.64 (m, 1H), 7.49 – 7.41 (m, 2H), 7.34 (d, *J* = 8.0 Hz, 1H), 7.20 (dd, *J* = 8.2, 7.0 Hz, 1H), 5.86 (d, *J* = 8.1 Hz, 1H), 3.88 (d, *J* = 7.9 Hz, 1H), 3.42 – 3.35 (m, 2H), 3.24 (d, *J* = 10.4 Hz, 1H), 2.84 (t, *J* = 12.1 Hz, 2H), 2.68 (s, 3H), 2.02 (d, *J* = 11.5 Hz, 4H), 1.73 (d, *J* = 12.1 Hz, 2H), 1.59 (t, *J* = 17.3 Hz, 2H), 1.46 (d, *J* = 12.1 Hz, 2H), 1.40 (s, 9H), 1.31 – 1.25 (m, 3H).

tert-Butyl (1-(5-(3-((*S*)-1-(4-fluorophenyl)ethyl)-1-((1*R*,4*S*)-4-(quinazolin-2-ylamino)cyclohexyl)ureido)-3-nitropyridin-2-yl)piperidin-4-yl) (methyl)carbamate (**14**).

Compounds **14** was prepared by a procedure similar to that used for compound **6d**. ^1^H NMOL/LR (500 MHz, DMSO-*d*_6_) *δ* 9.04 (s, 1H), 8.20 (d, *J* = 2.3 Hz, 1H), 7.96 (d, *J* = 2.3 Hz, 1H), 7.74 (dd, *J* = 8.0, 1.5 Hz, 1H), 7.63 (dd, *J* = 8.5, 6.8, 1.5 Hz, 1H), 7.38 (d, *J* = 8.5 Hz, 1H), 7.30 – 7.21 (m, 2H), 7.18 (dd, *J* = 8.0, 6.9, 1.1 Hz, 1H), 7.09 (t, *J* = 8.9 Hz, 2H), 6.28 (d, *J* = 8.1 Hz, 1H), 4.88 (*P*, *J* = 7.2 Hz, 1H), 4.24 (td, *J* = 11.8, 5.9 Hz, 1H), 3.89 (d, *J* = 13.0 Hz, 2H), 3.62 (d, *J* = 11.2 Hz, 1H), 3.09 (t, *J* = 12.6 Hz, 2H), 2.71 (s, 3H), 1.99 (s, 2H), 1.86 – 1.65 (m, 4H), 1.42 (s, 12H), 1.27 (d, *J* = 7.1 Hz, 3H), 1.10 (dd, *J* = 23.1, 11.4 Hz, 2H).

*tert-Butyl(1-(3-amino-5-(3-((S)-1-(4-fluorophenyl)ethyl)-1-((1R,4S)-4-(quinazolin-2-ylamino)cyclohexyl)ureido)pyridin-*2-yl*)piperidin-*4-yl*) (methyl)carbamate (****15****).* Compound **15** was prepared by a procedure similar to that used for compound **7a**. ^1^H NMOL/LR (500 MHz, DMSO-*d*_6_) *δ* 9.02 (s, 1H), 7.72 (d, *J* = 8.0 Hz, 1H), 7.60 (t, *J* = 7.8 Hz, 1H), 7.39 (d, *J* = 8.5 Hz, 1H), 7.34 (d, *J* = 2.3 Hz, 1H), 7.27 – 7.18 (m, 4H), 7.15 (t, *J* = 7.5 Hz, 1H), 7.08 (t, *J* = 8.7 Hz, 2H), 6.77 (d, *J* = 2.3 Hz, 1H), 5.54 (d, *J* = 8.1 Hz, 1H), 4.93 (s, 2H), 4.82 (q, *J* = 7.2 Hz, 1H), 4.17 (t, *J* = 13.7 Hz, 1H), 3.54 (dd, *J* = 20.5, 14.2 Hz, 3H), 2.72 (s, 3H), 2.65 (t, *J* = 12.4 Hz, 3H), 1.96 – 1.85 (m, 4H), 1.76 – 1.64 (m, 2H), 1.59 (s, 2H), 1.40 (s, 9H), 1.23 (t, *J* = 8.0 Hz, 5H), 1.14 – 1.04 (m, 2H).

*tert-Butyl(1-(3-(2-fluoroacrylamido)-5-(3-((S)-1-(4-fluorophenyl)ethyl)-1-((1R,4S)-4-(quinazolin-2-ylamino)cyclohexyl)ureido)pyridin-*2-yl*)piperidin-*4-yl*) (methyl)carbamate (****16****).* Compound **16** was prepared by a procedure similar to that used for compound **1d**. ^1^H NMOL/LR (500 MHz, DMSO-*d*_6_) *δ* 9.30 (d, *J* = 3.0 Hz, 1H), 9.04 (s, 1H), 7.97 – 7.90 (m, 2H), 7.74 (dd, *J* = 8.0, 1.5 Hz, 1H), 7.63 (t, *J* = 7.7 Hz, 1H), 7.39 (d, *J* = 8.5 Hz, 1H), 7.27 (td, *J* = 11.1, 9.9, 6.7 Hz, 3H), 7.17 (dd, *J* = 8.0, 6.9, 1.1 Hz, 1H), 7.09 (dd, *J* = 10.2, 7.6 Hz, 2H), 5.99 (d, *J* = 8.0 Hz, 1H), 5.85 – 5.73 (m, 1H), 5.53 (dd, *J* = 15.8, 3.8 Hz, 1H), 4.85 (*P*, *J* = 7.2 Hz, 1H), 4.23 (t, *J* = 11.9 Hz, 1H), 3.57 (d, *J* = 12.2 Hz, 3H), 2.91 – 2.83 (m, 2H), 2.74 (s, 2H), 1.97 (d, *J* = 10.1 Hz, 2H), 1.89 – 1.63 (m, 6H), 1.42 (s, 11H), 1.30 – 1.22 (m, 5H), 1.15 – 1.05 (m, 2H).

*N-(1-(3-(2-Fluoroacrylamido)-5-(3-((S)-1-(4-fluorophenyl)ethyl)-1-((1R,4S)-4-(quinazolin-2-ylamino)cyclohexyl)ureido)pyridin-*2-yl*)piperidin-*4-yl*)-N-methyl-1-(5-((3aS,4S,6aR)-2-oxohexahydro-1H-thieno[3,4-d]imidazole-*4-yl*)pentanamido)-3,6,9,12-tetraoxapentadecan-15-amide (****ZLC1223****).* To a solution of compound **16** (108 mg, 0.14 mmol) in anhydrous CH_2_Cl_2_ (2 mL) was added TFA (1 mL). The mixture was stirred for 1 h. Then the reaction was evaporated under reduced pressure. The residue was used directly in the next step without further purification. To a solution of the residue in DMF (10 mL) was added biotin-PEG4-propionic acid (64 mg), HATU (76 mg, 0.2 mmol) and DIPEA (68 μL). The resulting solution was stirred for 1 h. The solvent was evaporated under reduced pressure and purified using column chromatography to afford target **ZLC1223** (two steps, 90 mg, 56%). ^1^H NMOL/LR (600 MHz, DMSO-*d*_6_) *δ* 9.34 (d, *J* = 16.9 Hz, 1H), 9.04 (s, 1H), 7.97 – 7.90 (m, 2H), 7.83 (q, *J* = 6.0 Hz, 1H), 7.74 (dd, *J* = 8.0, 1.5 Hz, 1H), 7.62 (t, *J* = 7.6 Hz, 1H), 7.39 (d, *J* = 8.5 Hz, 1H), 7.31 – 7.22 (m, 3H), 7.17 (dd, *J* = 8.0, 6.9, 1.1 Hz, 1H), 7.11 – 7.06 (m, 2H), 6.45 – 6.38 (m, 1H), 6.35 (s, 1H), 5.99 (d, *J* = 7.9 Hz, 1H), 5.86 – 5.74 (m, 1H), 5.52 (dd, *J* = 15.7, 3.8 Hz, 1H), 4.86 (*P*, *J* = 7.2 Hz, 1H), 4.53 – 4.44 (m, 1H), 4.32 – 4.27 (m, 1H), 4.23 (td, *J* = 11.8, 5.9 Hz, 1H), 4.11 (dtd, *J* = 8.3, 4.1, 1.8 Hz, 1H), 3.67 – 3.62 (m, 2H), 3.59 (d, *J* = 12.1 Hz, 3H), 3.53 – 3.47 (m, 12H), 3.39 (q, *J* = 6.1 Hz, 2H), 3.18 (*P*, *J* = 5.7 Hz, 2H), 3.11 – 3.05 (m, 1H), 2.96 (t, *J* = 12.2 Hz, 1H), 2.88 (d, *J* = 7.3 Hz, 3H), 2.81 (dd, *J* = 12.4, 5.1, 3.2 Hz, 1H), 2.74 (s, 1H), 2.68 (t, *J* = 6.7 Hz, 1H), 2.60 – 2.55 (m, 2H), 2.08 – 2.03 (m, 2H), 2.00 – 1.88 (m, 3H), 1.82 (dd, *J* = 12.1, 4.1 Hz, 2H), 1.74 (d, *J* = 12.0 Hz, 2H), 1.60 (dq, *J* = 12.5, 7.3, 4.5 Hz, 2H), 1.53 – 1.36 (m, 5H), 1.35 – 1.19 (m, 7H), 1.11 (dq, *J* = 25.4, 12.4 Hz, 2H). ^13^C NMOL/LR (151 MHz, DMSO) *δ* 172.13, 169.99, 169.88, 162.71, 162.03, 161.60, 160.00, 158.86, 157.54, 157.33, 156.71, 156.02, 154.93, 153.59, 153.58, 151.59, 145.76, 142.00, 134.81, 133.36, 133.21, 129.66, 129.18, 129.12, 127.86, 124.77, 124.10, 124.02, 121.72, 119.51, 115.89, 114.58, 101.91, 99.67, 69.81, 69.74, 69.58, 69.18, 67.17, 66.80, 61.04, 59.20, 55.44, 53.70, 53.65, 50.05, 48.91, 48.87, 48.52, 38.45, 35.10, 33.77, 33.08, 31.24, 30.97, 30.40, 30.28, 29.60, 29.36, 28.63, 28.20, 28.05, 26.82, 25.27, 22.44, 22.08, 11.82. HRMS (ESI) for C_58_H_78_F_2_N_12_NaO_9_S [M+Na]^+^, calcd: 1179.5696, found: 1179.5606. HPLC purity: 96.90%.

### 4.2 Animals

#### 4.2.1 Pharmacokinetics study

The pharmacokinetic investigation was conducted by Medicilon Inc (Shanghai, China) and was ethically approved by Medicilon Institutional Animal Care and Use Committee (26048-24003-NG). Sprague–Dawley male rats (specific pathogen free) were dosed with compound solution (5% DMSO, 10% Solutol and 85% saline) by both intravenous injection (2 mg/kg) and oral administration (10 mg/kg). The collection and the following processing of blood samples, as well as the calculation of pharmacokinetic parameters, were performed as previously described^12^^,^^13^.

#### 4.2.2 Drug toxicity and MTD test in CD1 mouse

The experiments were performed under an approved animal protocol by the Institutional Animal Care & Use Committee of the University of Michigan, Ann Arbor, USA. Eight-week-old CD-1 male and female mice were obtained from Jackson Lab for toxicity testing. The animals were treated with vehicle, 2, 5, 10, and 20 mg/kg once daily for three weeks. Body weight was recorded daily.

##### 4.2.2.1 Complete blood cell count

Blood samples were collected from submandibular bleed and directly transferred to K3 EDTA tubes to prevent coagulation. Following collection, the blood samples were immediately mixed with EDTA by gentle inversion. Complete blood count (CBC) was performed by University of Michigan *in vivo* animal core (IVAC) on the day of blood collection to maintain sample integrity.

##### 4.2.2.2 Serum chemistry analysis

Serum for biochemical analysis was obtained through a standardized collection and separation process. About 200 μL of blood was drawn into serum separator tubes and incubated for 20 min at R.T. to allow for clotting. Tubes were then centrifuged at 1800–3000×*g* for 10 min. The cell-free serum was then extracted from the tubes for analysis at IVAC.

##### 4.2.2.3 Histopathological analysis of organs

Multiple organs, including the liver, spleen, kidney, colon, small intestine, mesenteric lymph nodes, ovaries, prostate, and testis, were systematically evaluated for histopathological changes. Two pathologists, blinded to the control and treatment groups, examined the stained sections under a brightfield microscope. They assessed general tissue morphology and architectural coherence across all organs. Detailed analyses were conducted at cellular and sub-cellular levels, focusing on specific aspects unique to each organ.

#### 4.2.3 *In vivo* efficacy study

Animal experiments were performed under an approved animal protocol by the Institutional Animal Care & Use Committee of the University of Michigan, Ann Arbor, USA. Briefly, 3 × 10^6^ VCaP cells were resuspended in serum-free media and mixed with 50% Matrigel (BD Biosciences, Franklin Lakes, USA), then subcutaneously injected into both dorsal flanks of six-to-eight-week-old CB17SCID male mice (Charles River Laboratory, Boston, USA). Upon tumors reaching an approximate volume of 200 mm^3^, animals underwent castration to induce tumor regression. When tumors subsequently regrew to their pre-castration size, mice were randomized and treated with 2 mg/kg q.d., 5 mg/kg q.d. And 10 mg/kg q.o.d. **ZLC853** or vehicle by oral gavage over 3–4 weeks. Vehicle consisted of 20% PEG400, 6% Cremophor EL, and 74% PBS solution. Tumors were collected at the end of the experiment for qPCR, Western blot and histology analysis.

#### 4.2.4 PDX study

The LTL706B (*CDK12*-mutant) tumor was obtained from the Vancouver Prostate Centre and initially established in the renal capsule of NSG mice with a testosterone pellet (12.5 mg) implant. Once tumors grew successfully, we transferred them into subcutaneous pockets of CB17SCID mice for therapy studies. LuCaP23.1 (*CDK12*-intact) PDX tumors were obtained from the University of Washington. Tumors from LuCaP23.1 PDX lines were maintained subcutaneously in dorsal flanks of CB17SCID male mice.

### 4.3 *In vitro* kinase enzyme assay

The kinase activity detection and the KinomeScan selectivity profiles were performed using an ADP-Glo kinase assay by ICE Bioscience Inc (Beijing, China).

### 4.4 Protein expression and purification

Proteins used for crystallization and mass spectrometry analysis were expressed in *Spodoptera frugiperda* cells (Sf9) transfected with codon-optimized synthetic genes comprising the kinase domain of human CDK12 (UniProt accession number Q9NYV4, residues 715-1052), the kinase domain of human CDK13 (UniProt accession number Q14004, residues 694-1039), the cyclin box domain of human Cyclin K (UniProt accession number O75909, residues 1-267), and full-length CAK1 from *Saccharomyces cerevisiae* (UniProt accession number P43568, residues 1−368). CDK12 and cyclin K or CDK13 and cyclin K were fused with an N-terminal hexahistidine (6 × His) tag followed by a tobacco etch virus (TEV) protease cleavage site and were co-cloned into a pFastBac dual expression vector, while CAK1 was cloned into a pFastBac 1 expression vector using the Invitrogen Baculovirus Expression System. DNA was prepared in *Escherichia coli* strain DH10Bac and used to generate baculovirus in Sf9 insect cells. Baculoviruses of CDK12/cyclin K or CDK13/cyclin K and CAK1 were used to co-infect Sf9 cells. The cultures were centrifuged at room temperature for 10 min at 13,900×*g* after 72 h of infection.

The cell pellet was suspended in lysis buffer (25 mmol/L HEPES, 300 mmol/L NaCl, 1 mmol/L TCEP, pH 7.5) and lysed by a high-pressure homogenizer. The cell debris was then removed by centrifugation at 38,759×*g* at 4 °C for 40 min. Recombinant proteins in the supernatant were purified using nickel-sepharose resin (Cytiva, Marlborough, USA) and eluted stepwise with imidazole. Fractions containing the CDK12/cyclin K or CDK13/cyclin K complex were treated with TEV protease for cleavage of the 6 × His tags. Proteins were further purified using the reverse nickel-affinity method. For final purification, proteins were applied to a Superdex 75 size-exclusion column (Cytiva, Marlborough, USA) equilibrated with storage buffer (25 mmol/L HEPES, 150 mmol/L NaCl, 2 mmol/L DTT, pH 7.5). Proteins were pooled, concentrated to 10 mg/mL, flash-frozen in liquid nitrogen, and stored at −80 °C.

### 4.5 Cell culture

Cells used in this study were purchased from American Type Culture Collection (ATCC) or Chinese Academy of Sciences National Collection of Authenticated Cell Cultures, and cultured according to the corresponding guidelines at 37 °C in 5% CO_2_ incubator. All media were supplemented with 10% fetal bovine serum (FBS), 100 U/mL penicillin, and 100 μg/mL streptomycin. DMEM for VCaP cell culture was additionally supplemented with GlutaMAX. Cell lines were tested for mycoplasma using the Lonza MycoAlert Kit (Lonza, Basel, Switzerland) following the manufacturer's protocol.

### 4.6 Cell proliferation assay

Tested cells were seeded in 96-well or 384-well plates and incubated overnight, following with the addition of a series of compound dilutions. Cell proliferation was measured 3 days after compound treatment using Cell Counting Kit-8 (Selleck, Shanghai, China) according to the manufacturer's instructions. The absorbance signal at OD_450_ and OD_650_ was detected by EnVision plate reader (Revvity, Waltham, USA), and the IC_50_ values were determined by nonlinear regression and a four-parameter algorithm using GraphPad Prism software (San Diego, USA).

### 4.7 Western blotting

The Western blotting was performed as previously described^11^^,^^13^. After treatment under various conditions, cell lysates were prepared using RIPA buffer (Thermo Scientific, Waltham, USA) supplemented with complete Protease Inhibitor Cocktail Tablets (Sigma–Aldrich, St. Louis, USA). Protein concentrations were determined using the Pierce BCA Protein Assay Kit (Bio-Rad, Hercules, USA). Equal amounts of protein from each sample were loaded onto NuPAGE 3%–8% Tris-Acetate or 4%–12% Bis-Tris protein gels (Thermo Scientific, Waltham, USA), followed by electrophoretic separation and transfer to membranes. Membranes were then blocked with 5% non-fat milk in PBST buffer (0.1% Tween-20) for 1 h at room temperature. After blocking, membranes were incubated with appropriate primary antibodies overnight at 4 °C. HRP-conjugated secondary antibodies were used for detection, and protein bands were visualized using the Odyssey Fc Imaging System (LI-COR Biosciences, Lincoln, USA).

### 4.8 Pull-down assay

Briefly, VCaP cells were pretreated with **ZLC853** or **ZLC1178** for 2 h, followed by the washout and subsequent incubation with **ZLC1223** for an additional 4 h. After treatment, cells were lysed in RIPA buffer (Thermo Scientific, Waltham, USA), and the resulting cell lysates were incubated with High-Capacity Neutravidin Agarose Resin (Thermo Scientific, Waltham, USA) overnight at 4 °C. The beads were washed with PBST buffer (0.1% Tween-20) three times and eluted with 2 × SDS loading buffer. The eluted protein samples were then subjected to Western blot analysis.

### 4.9 Wash-out assay

VCaP cells were treated with 300 nmol/L of **ZLC853**, **ZLC1178**, or DMSO for 2 h. For the wash-out groups, cells were thoroughly washed with PBS to remove the compounds. The DMSO control group remained untreated beyond the initial exposure period, as described above. After washing, cells in the wash-out groups were further cultured and harvested at various time points. Collected cells were lysed and subjected to Western blot analysis to evaluate protein expression changes over time.

### 4.10 Real-time quantitative PCR assay

Total RNA was extracted from VCaP cells using the RNeasy Mini Kit (Qiagen, 74,104) according to the manufacturer's instructions. RNA concentration and purity were assessed using the NanoDrop 2000 spectrophotometer (Thermo Scientific, Waltham, USA). A total of 1000 ng RNA was used for cDNA synthesis using the Maxima First Strand cDNA Synthesis Kit for RT-PCR (Thermo Scientific, Waltham, USA). Quantitative real-time PCR (qPCR) was performed in triplicate using SYBR Green master mix on QuantStudio 6 Pro Real-Time PCR System (Applied Biosystems, Foster City, USA). Gene expression levels were quantified using the *Δ*Ct method and normalized to ACTB expression. Primers for qPCR were synthesized by Integrated DNA Technologies, and the sequences are listed in Supporting Information Table S4.

### 4.11 Protein digestion and HPLC–tandem MS (MS/MS) analysis of peptides

Proteins were precipitated with acetone. The protein pellet was dried by using a Speedvac for 1−2 min. The pellet was subsequently dissolved in 8 mol/L urea, 100 mmol/L Tris–HCl, pH 8.5. TCEP (final concentration is 5 mmol/L) and Iodoacetamide (final concentration is 10 mmol/L) for reduction and alkylation were added to the solution and incubated at room temperature for 30 min, respectively. The protein mixture was diluted four times and digested overnight with Trypsin at 1:50 (*w*/*w*) (Promega, Madison, USA). The digested peptides were desalted using a home-packed C18 column and dried with a SpeedVac.

The peptide mixture was analyzed by a home-made 25 cm-long pulled-tip analytical column (75 μm ID packed with ReproSil-Pur C18-AQ 1.9 μm resin, Dr. Maisch GmbH), and the column was then placed in-line with an Easy-nLC 1200 nano HPLC (Thermo Scientific, Waltham, USA) for mass spectrometry analysis. The analytical column temperature was set at 55 °C during the experiments. The mobile phase and elution gradient used for peptide separation were as follows: 0.1% formic acid in water as buffer A and 0.1% formic acid in 80% acetonitrile as buffer B, 0–1 min, 2%–10% B; 1–81 min, 10%–35% B; 81–96 min, 35%–60% B; 96–111 min, 60%–100% B; 111–120 min, 100% B. The flow rate was set as 300 nL/min.

### 4.12 Mass spectrometry and data analysis

Data-dependent MS/MS analysis was performed with a Q Exactive mass spectrometer (Thermo Scientific, Waltham, USA). Peptides eluted from the LC column were directly electrosprayed into the mass spectrometer with the application of a distal 2.2-kV spray voltage. A cycle of one full-scan MS spectrum (*m*/*z* 300–1800) was acquired, followed by the top 20 MS/MS events, sequentially generated on the first to the twentieth most intense ions selected from the full MS spectrum at a 28% normalized collision energy. Full scan resolution was set to 70,000 with an automated gain control (AGC) target of 3e6. MS/MS scan resolution was set to 17,500 with an isolation window of 1.8 *m*/*z* and AGC target of 5e5. The number of microscans was one for both MS and MS/MS scans, and the maximum ion injection time was 50 and 100 ms, respectively. The dynamic exclusion settings used were as follows: charge exclusion, 1 and > 7; exclude isotopes, on; and exclusion duration, 15 s. MS scan functions and LC solvent gradients were controlled by the Xcalibur data system (Thermo Scientific, Waltham, USA).

The acquired MS/MS data were analyzed against a Swiss-Prot *Homo Sapiens* database using PEAKS Studio 8.5 (Bioinformatics Solutions, Waterloo, Canada). Precursor and product ion spectra were searched with an initial mass tolerance of 20 ppm and 0.02 Da, respectively. Semi-specific trypsin cleavage was selected, and up to three missed cleavages were allowed. Compound covalent binding on cysteine, phosphorylation on serine or threonine or tyrosine, carbamidomethylation on cysteine, and oxidation on methionine were set as variable modifications. FDR was set as 1% and protein identification threshold was set as (−10 lg*P*) ≥20. MSMS spectra of the modified peptides were manually confirmed and annotated.

### 4.13 Crystallography

The purified CDK12/CCNK protein complex was stored in a buffer of 25 mmol/L HEPES, pH 7.5, 150 mmol/L NaCl and 2 mmol/L DTT. Prior to setting up crystallization trials, compound diluted in DMSO was added in a 1:5 ratio to the pooled kinase complexes (3.8 mg/mL) and incubated for 30 min on ice, in order to generate a CDK12/CCNK/compound ternary complex. Crystallization conditions were screened through Morpheus kit (Molecular Dimensions, Sheffield, UK) using the hanging drop vapor diffusion technique by mixing the protein solution in a 1:1 ratio with the reservoir solution. Big single crystals were obtained at 291 K in condition F2, containing 10% *w*/*v* PEG 8000, 20% *v*/*v* ethylene glycol, 0.02 mol/L of each monosaccharide, 0.1 mol/L MES/imidazole (pH 6.5). The crystals were rapidly flash-frozen in ALS-style pucks that were submerged in liquid nitrogen. The diffraction data were collected at Protein Microcrystal Structure beamline 18U (Shanghai Synchrotron Radiation Facility, Shanghai, China) and processed using the XDS software package, pointless and aimless. The complex structure of compound **ZLC853** with CDK12/CCNK was determined by molecular replacement using the program PHASER in the CCP4 suite. An iterative cycle of refinement was carried out using COOT and PHENIX. Structure figures were created using PyMol (Schrödinger, Inc, New York City, USA).

### 4.14 Cell permeability study

The Cell permeability evaluation of **ZLC853** was evaluated in Medicilon Inc (Shanghai, China). Compound **ZLC853** was diluted with the transport buffer (HBSS with BSA) from a 10 mmol/L stock solution to a concentration of 10 μmol/L and applied to the apical or basolateral side of the cell monolayer. Permeation of the compound **ZLC853** from A to B direction or B to A direction was determined in duplicate over a 120-min incubation at 37 °C and 5% CO_2_ with a relative humidity of 95%. The efflux ratio of each compound was also determined. Test and reference compounds were quantified by LC–MS/MS analysis based on the peak area ratio of analyte/IS.

### 4.15 Plasma protein binding study

The plasma protein binding evaluation of **ZLC853** was conducted in Medicilon Inc (Shanghai, China). The equilibrium dialysis method was utilized to detect the protein-binding ratio of **ZLC853** in rat plasma. The protocol of the experiment is as follows: (1) Preheat PBS buffer, pH 7.4; (2) Prepare plasma: (a) Thaw frozen plasma by placing at 37 °C; (b) Centrifuge plasma at 13,523×*g* for 5 min to remove clots, pipette and pool the supernatant as the plasma to be used in the experiment; (3) Pretreat membrane: Dialysis membrane strips were soaked in distilled water for an h. Add 20% by of volume ethanol and soak for a further 20 min. The membrane strips were then rinsed in distilled water 3 times before use. (4) Spike solutions of test and reference compounds: Solution A (0.5 mmol/L): Add 5 μL of 10 mmol/L stock solution into 95 μL of DMSO. (5) Prepare the test and reference compounds in plasma: (a) Preload a 96-well plate with 495 μL aliquots of plasma in the wells designated for plasma, respectively. (b) Spike 5 μL of Solution A, 0.5 mmol/L of test and reference compounds into the pre-loaded plasma in the 96-well plate. The final test concentration is 5 μmol/L (6) Dialyze Sample Loading: (a) Apply aliquots of 100 μL of blank dialysis buffer to the receiver side of dialysis chambers. Then apply aliquots of 100 μL of the plasma spiked with test and reference compounds to the donor side of the dialysis chambers. (b) Aliquot 25 μL of the plasma spiked with test and reference compounds into a 96-well sample preparation plate as T0 samples and store the sample plate in a freezer (−20 °C). Mix the plasma samples (from step 5. b) with the same volume of blank buffer (25:25, *v*/*v*). Quench the samples with 200 μL of ACN:MeOH (1:1) containing internal standard (IS). Vortex the samples at 49×*g* for 10 min, cover the plate and store it in a freezer (−20 °C). (c) Cover the dialysis block with a plastic lid and place the entire apparatus in a shaker (4.9×*g*) for 5 h at 37 °C. (d) Aliquot 25 μL from both the donor sides and receiver sides of the dialysis apparatus into new sample preparation plates and mix the aliquots with the same volume of opposite matrices. Quench the samples with 200 μL ACN:MeOH (1:1) containing IS. Vortex all the samples (from 0 to 5 h) at 49×*g* for 10 min, followed by centrifugation at 490×*g* for 15 min. Transfer 100 μL of the supernatant from each well into a 96-well sample plate containing 100 μL of ultra-pure water for LC–MS analysis.

### 4.16 hERG inhibition study

The hERG channel inhibition of **ZLC853** was evaluated in HEK293 cells that expressed hERG using a manual patch-clamp technique. In brief, **ZLC853** was tested at 0.3, 1, 3, 10 and 30 μmol/L concentrations, with terfenadine included as the positive control. The compounds were evaluated at room temperature by a PatchMaster patch-clamp system.

### 4.17 CYP inhibition study

A PBS buffer system containing 0.2 mg/mL human liver microsomes was used to evaluate the inhibitory effect of compounds on eight common CYP450 enzymes (CYP1A2, CYP2B6, CYP2C8, CYP2C9, CYP2C19, CYP2D6, and CYP3A4 (Midazolam/Testosterone)). The concentrations of the compounds were 10.00, 3.33, 1.11, 0.37, 0.12, 0.04, and 0.01 μmol/L. In the reaction system, the substrates of CYP1A2, CYP2B6, CYP2C8, CYP2C9, CYP2C19, CYP2D6, and CYP3A4 were phenacetin (30 μmol/L), bupropion/Amfebutamone HCl (70 μmol/L), paclitaxel (10 μmol/L), diclofenac (10 μmol/L), mephenytoin (35 μmol/L), dextromethorphan (5 μmol/L), and midazolam/testosterone (5/80 μmol/L), respectively. UPLC-MS/MS was used to detect the metabolites of the positive substrates to calculate the IC_50_ values of the evaluated compounds for each enzyme.

### 4.18 Metabolite identification assay

ZLC853 at a final concentration of 10 μmol/L was individually incubated with mouse (ICR), rat (SD), dog (Beagle), monkey (Cynomolgus) and human (mixed gender) liver microsomes (1 mg/mL, purchased from IPHASE) in 1 mL PBS buffer containing 3 mmol/L MgCl_2_ and 1 mmol/L NADPH at 37 °C for 0 or 60 min. Incubations were quenched with three volumes of pre-cooling methanol-acetonitrile (50:50, *v*/*v*) followed by centrifugation for 5 min at 13,000×*g*. 90% of the supernatant was collected and evaporated using a low-temperature vacuum concentrator. The dried samples were redissolved in 50 μL water-acetonitrile (30:70, *v*/*v*) and 100 μL water, followed by centrifugation for 10 min at 12,000×*g*. Supernatant was then analyzed by UPLC-UV–HRMS.

### 4.19 CYP phenotyping determination

Substrates specific to different CYP isoforms were prepared as working solutions at the following concentrations: phenacetin for CYP1A2: 75 mmol/L, bupropion for CYP2B6: 60 mmol/L, amodiaquine for CYP2C8: 1 mmol/L, diclofenac for CYP2C9: 8 mmol/L, *s*-mephenytoin for CYP2C19: 20 mmol/L, dextromethorphan for CYP2D6: 4 mmol/L, midazolam for CYP3A: 2 mmol/L. Inhibitors specific to different CYP isoforms were prepared as working solutions at the following concentrations: Furafylline for CYP1A2: 2 mmol/L, Ticlopidine for CYP2B6: 0.6 mmol/L, Montelukast for CYP2C8: 0.5 mmol/L, Sulfaphpenazole for CYP2C9: 0.5 mmol/L, *N*-3-benzylnirvanol for CYP2C19: 0.5 mmol/L, Quinidine for CYP2D6: 0.2 mmol/L, Ketoconazole for CYP3A: 0.2 mmol/L. **ZLC853** and NADPH were prepared as working solutions at 1 and 10 mmol/L, respectively. Prepare the master solution by adding two μL of the substrate/test compound and 50 μL of pre-warmed human liver microsomes into 948 μL PBS buffer. All samples were incubated in a 37 °C water bath, with three parallels for each concentration of the test compound and three parallels of the positive control inhibitor. Take 100 μL of microsome working solution, add two μL of inhibitor working solution, add two μL of solvents to the solvent control, and put it in a water bath to pre-incubate for 5 min. After pre-incubation, add 98 μL of NADPH regeneration solution to all samples to start the reaction. Then put it back in the water bath and incubate for a certain period of time. After a certain period of incubation. The reaction was stopped by the addition of 200 μL of cold acetonitrile with IS (50 ng/mL tolbutamide). Samples were centrifuged at 1503×*g* for 10 min. Aliquot of 100 μL of the supernatant was mixed with 100 μL of ultra-pure H_2_O and then used for LC–MS/MS analysis.

### 4.20 Histology analysis

H&E staining of formalin-fixed paraffin-embedded (FFPE) tissue sections and histological assessment were performed as previously described^11^, ^12^, ^13^.

### 4.21 Statistical analysis

All data points were acquired with biological replicate samples. Data were analyzed and plotted with Prism version 10 (GraphPad Software; San Diego, CA) and presented as means ± standard deviation (SD) or ± standard error of mean (SEM), as stated in the figure legends. To determine statistical differences, Student's *t* test was used for individual comparisons, one-way ANOVA for multiple comparisons, and two-way ANOVA for multiple variables. Statistical significance was determined with a *P*-value less than 0.05, unless otherwise stated.

## Author contributions

Licheng Zhou, Kaijie Zhou, Jean C. Tien, Zhen Wang, George Xiaoju Wang, Arul M. Chinnaiyan, and Ke Ding conceived the study and designed the research. Kaijie Zhou: Methodology, Validation, Formal analysis, Investigation, Data Curation, Writing - Original Draft, Visualization. Licheng Zhou: Conceptualization, Methodology, Investigation, Formal analysis, Data Curation, Writing - Original Draft. Jean C. Tien: Methodology, Validation, Formal analysis, Investigation, Data Curation, Writing - Review & Editing. Wenyan Liu, Xinyu Sun, Bohai Fan, Xin Huang, Jun Wang, Yu Chang, Jianzhang Yang, Yue Yin, Chen Su, Qingrun Li, Rahul Mannan, Somnath Mahapatra, Yunhui Cheng, Abigail J. Todd, Christina E. Wheeler, Shuqin Li, Yang Zhou, Xiaomei Ren, Validation, Investigation. George Xiaoju Wang: Methodology, Formal analysis, Investigation. Weixue Huang and Zhen Wang: Formal analysis, Writing - Review & Editing, Project administration. Arul M. Chinnaiyan, and Ke Ding: Conceptualization, Supervision, Project administration, Writing - Review & Editing, Funding acquisition.

## Conflicts of interest

The authors declare the following competing financial interest(s): Shanghai Institute of Organic Chemistry and the University of Michigan filed patent applications on these CDK12/CDK13 covalent inhibitors described in this study, in which Ke Ding, Licheng Zhou, Arul M. Chinnaiyan, Kaijie Zhou, Jianzhang Yang, Bohai Fan, Yu Chang, Zhen Wang, Weixue Huang, George Xiaoju Wang, and Jean C. Tien are named as inventors. The work is partially supported by Livzon Pharmaceutical Group Inc., Zhuhai City, China.

## Appendix A Supporting information

The following is the Supporting Information to this article.
