# Supplementary material for: Discovery and preclinical evaluation of ZLC853 as a potential clinical candidate covalently targeting CDK12/13 kinases
Source: Acta Pharm Sin B. 2026 Jul 29;16(11):7554–72. doi: 10.1016/j.apsb.2026.07.041 (PMC13729719; doi:10.1016/j.apsb.2026.07.041)
Supplement: Supplementary file 1 [file mmc1.docx]

**Supporting information for**

**Original article**

**Discovery and preclinical evaluation of ZLC853 as a potential clinical candidate covalently targeting CDK12/13 kinases**

**Kaijie Zhou^a,b,^**^†^**, Licheng Zhou^a,c,^**^†^**, Jean C. Tien^d,e,^**^†,^**^*^, Weixue Huang^a,^**^†^**, Wenyan Liu^a,d^, Xinyu Sun^a^, Bohai Fan^a^, Xin Huang^a,b^, Jun Wang^a,b^, Yu Chang^a,d,e^, Jianzhang** **Yang^a,c^, Yue Yin^f^, Chen Su^f^, Qingrun Li^f^, Rahul Mannan^d,e^, Somnath Mahapatra^d,e^, Yunhui Cheng^d,e^, Abigail J. Todd^d,e^, Christina E. Wheeler^d,e^, Shuqin Li^d,e^, Yang Zhou^c^, Xiaomei Ren^a^, George Xiaoju Wang^d,e,h,*^, Zhen Wang^a,*^, Arul M. Chinnaiyan^d,e,g,h,I,*^, Ke Ding^a,b,*^**

^a^State Key Laboratory of Chemical Biology, Shanghai Institute of Organic Chemistry, Chinese Academy of Sciences, Shanghai 200032, China

^b^University of Chinese Academy of Sciences, Beijing 101408, China

^c^International Cooperative Laboratory of Traditional Chinese Medicine Modernization and Innovative Drug Discovery of Chinese Ministry of Education (MOE), Guangzhou City Key Laboratory of Precision Chemical Drug Development, College of Pharmacy, Jinan University, Guangzhou 511400, China

^d^Michigan Center for Translational Pathology, University of Michigan, Ann Arbor, MI 48109, USA

^e^Department of Pathology, University of Michigan, Ann Arbor, MI 48109, USA

^f^National Facility for Protein Science in Shanghai, Shanghai Advanced Research Institute, Chinese Academy of Science, Shanghai 201210, China

^g^Howard Hughes Medical Institute, University of Michigan, Ann Arbor, MI 48109, USA

^h^Rogel Cancer Center, University of Michigan, Ann Arbor, MI 48109, USA

^i^Department of Urology, University of Michigan, Ann Arbor, MI 48109, USA

Received 1 November 2025; received in revised form 18 February 2026; accepted 16 March 2026

^†^These authors contributed equally to this work.

*Corresponding authors.

E-mail addresses: jtien@med.umich.edu (Jean C. Tien), xiaojuw@med.umich.edu (Xiaoju Wang), wangz@sioc.ac.cn (Zhen Wang), arul@med.umich.edu (Arul M. Chinnaiyan), dingk@sioc.ac.cn (Ke Ding).

**Supplementary Figures**

**Figure S1** Predicted metabolites of **YJZ5118.** Metabolites are predicted by a publicly available program (SMARTCyp) and medicinal chemistry knowledge.

**Figure S2** Covalent binding validation of ZLC853. (A, B) Mass spectral peaks of Cyclin K in CDK12/Cyclin K (A) or CDK13/Cyclin K (B) protein complex treated with DMSO (top) or a 5-fold excess of **ZLC853** (bottom) overnight at room temperature. (C, D) MS/MS spectrum of CDK12 peptide ^1033^LPHWQDC HELWSK^1045^ (C) and CDK13 peptide ^1008^PPDLPLWQDC HELWSK^1023^ (D) modified with **ZLC853** indicates covalent labeling of Cys1039 and Cys1017. Types b, y ions and inhibitor related ions are indicated in blue, red and green, respectively.

**Figure S3** Results of the pulldown assay. VCaP cells were incubated with 3 μmol/L of biotin or ZLC1223 for 4 h.

**Figure S4** Main detected metabolites of ZLC853. *N*-Dealkylation product (M13) was identified as the predominant metabolite in liver microsomes across all species tested (mouse, rat, dog, monkey, and human). Notably, in human and rat liver microsomes, M13 was essentially the exclusive metabolite detected. Additionally, primary oxidative metabolites (M6, M8, and M12) resulting from mono-oxygenation were observed in mouse, dog, and monkey liver microsomes.

**Figure S5** Body weight, organ weight and H&E staining results in the *in vivo* toxicity investigation of ZLC853. (A) Percent body weight change in CD-1 mice treated with ascending concentrations of **ZLC853**. Data are presented as mean ± SD (*n* = 5, biological replicates). (B) Organ weight from CD-1 mice described in A. Data are presented as mean ± SD (*n* = 3–5, biological replicates). **P* ≤ 0.05, ***P* ≤ 0.01, ****P* ≤ 0.001, *****P* ≤ 0.0001 by *t*-test. (C) Representative H&E staining of liver, kidney, spleen, and small intestine from mice treated with indicated **ZLC853** concentrations.

**Figure S6** Complete blood count and liver and kidney function tests in the *in vivo* toxicity investigation of ZLC853. (A) Complete blood count from CD-1 mice described in A. Data are presented as mean ± SD (*n* = 3–5, biological replicates). **P* ≤ 0.05, ***P* ≤ 0.01, ****P* ≤ 0.001, *****P* ≤ 0.0001 by *t*-test. (B) Liver and kidney function tests from **ZLC853**-treated CD-1 mice. ALB: albumin; ALT: alanine transaminase; TBIL: total bilirubin; CREA: creatinine; BUN: blood urea nitrogen).

**Supporting tables**

**Table S1** Data collection and refinement statistics (molecular replacement)

|  | **CDK12/Cyclin K/ZLC853** |
| --- | --- |
| PDB ID | 9X26 |
| **Data collection** |  |
| Wavelength(Å) | 0.979 |
| Space group | *P* 3_1_ 2 1 |
| Cell dimensions |  |
| *a*, *b*, *c* (Å) | 114.12, 114.12, 103.06 |
| *α, β, γ* (°) | 90.00, 90.00, 120.00 |
| Resolution (Å) | 37.35–3.28 (3.54–3.28) * |
| *R*_merge_ | 0.115 (1.029) |
| *I/σ(I)* | 18.3 (3.6) |
| CC^1/2^ | 0.999 (0.960) |
| Completeness (%) | 99.9 (99.9) |
| Redundancy | 18.7 (19.2) |
|  |  |
| **Refinement** |  |
| Resolution (Å) | 37.35–3.28 |
| No. Unique reflections | 12214 |
| *R*_work_/*R*_free_ | 0.1970/0.2470 |
| No. atoms | 4478 |
| Protein | 4425 |
| Ligand/ion | 51 |
| Water | 2 |
| *B*-factors | 131.1 |
| Protein | 131.1 |
| Ligand/ion | 133.0 |
| Water | 66.6 |
| R.m.s. deviations |  |
| Bond lengths (Å) | 0.0069 |
| Bond angles (°) | 1.5670 |
| Ramachandran plot  Most favored region (%)  Allowed regions (%)  Disallowed regions (%) | 94.4  4.9  0.7 |

*Values in parentheses are for highest-resolution shell.

**Table S2** KinomeScan Profiling Data for Compound **ZLC853**

| Kinase | ZLC853 (1 μmol/L) | ZLC853 (1 μmol/L) | STDEV | ZLC853  (%inhibition)@1 μmol/L |
| --- | --- | --- | --- | --- |
| JNK1 | 111.36 | 101.12 | 7.24 | 106.24 |
| JNK2 | 103.77 | 105.90 | 1.51 | 104.83 |
| JNK3 | 102.11 | 101.93 | 0.13 | 102.02 |
| CDK12/CyclinK | 101.54 | 97.23 | 3.05 | 99.38 |
| CDK13/CyclinK | 98.11 | 97.98 | 0.10 | 98.05 |
| TAOK1 | 92.79 | 91.58 | 0.86 | 92.19 |
| CDK7/CycH/MAT1 | 90.62 | 90.76 | 0.10 | 90.69 |
| CDK6/CyCD3 | 71.98 | 70.62 | 0.96 | 71.30 |
| TAOK2 | 72.12 | 66.53 | 3.95 | 69.32 |
| CDK6/CycD1 | 67.06 | 70.28 | 2.28 | 68.67 |
| TAOK3 | 67.40 | 68.43 | 0.73 | 67.92 |
| DYRK1B | 66.66 | 64.14 | 1.78 | 65.40 |
| SGK | 62.53 | 64.04 | 1.07 | 63.29 |
| DYRK2 | 59.25 | 65.98 | 4.76 | 62.61 |
| CDK5/p25NCK | 63.43 | 58.11 | 3.76 | 60.77 |
| Erk7 | 52.61 | 54.20 | 1.13 | 53.41 |
| CDK9/CycT1 | 51.30 | 51.07 | 0.16 | 51.19 |
| CK1*δ* | 44.49 | 53.27 | 6.21 | 48.88 |
| CK1*γ*1 | 52.82 | 44.77 | 5.70 | 48.80 |
| CDK18/CyclinY | 49.30 | 47.71 | 1.13 | 48.51 |
| CK1*γ*2 | 54.92 | 41.39 | 9.57 | 48.16 |
| CDK9/CycT2 | 43.75 | 48.98 | 3.69 | 46.36 |
| Erk5 | 45.75 | 44.83 | 0.65 | 45.29 |
| CDK4/CycD3 | 38.42 | 50.74 | 8.71 | 44.58 |
| TTK | 47.00 | 40.53 | 4.57 | 43.77 |
| CDK5/p35NCK | 49.11 | 34.11 | 10.61 | 41.61 |
| DYRK1A | 41.64 | 40.04 | 1.13 | 40.84 |
| PKC*β*2 | 36.64 | 36.16 | 0.33 | 36.40 |
| PHKG1 | 39.58 | 30.60 | 6.35 | 35.09 |
| CDK16/CyclinY | 37.54 | 28.06 | 6.71 | 32.80 |
| CDK9/CycK | 22.41 | 39.29 | 11.93 | 30.85 |
| CDK1/CycA2 | 28.94 | 32.57 | 2.57 | 30.75 |
| MET D1228N | 31.61 | 29.84 | 1.25 | 30.73 |
| MER | 22.60 | 36.59 | 9.89 | 29.59 |
| PKD1 | 19.11 | 35.68 | 11.72 | 27.39 |
| MET Y1230A | 30.45 | 22.65 | 5.51 | 26.55 |
| PRKCZ | 29.92 | 22.82 | 5.02 | 26.37 |
| CDK4/CycD1 | 24.76 | 26.86 | 1.49 | 25.81 |
| PKD2 | 23.52 | 27.78 | 3.01 | 25.65 |
| PKN2 | 22.23 | 28.40 | 4.36 | 25.32 |
| MET Y1230C | 30.14 | 20.05 | 7.14 | 25.10 |
| SGK2 | 25.99 | 23.25 | 1.94 | 24.62 |
| CDK1/CycE1 | 22.03 | 26.91 | 3.45 | 24.47 |
| SGK3 | 29.79 | 17.33 | 8.81 | 23.56 |
| DDR1 | 16.61 | 30.34 | 9.71 | 23.47 |
| CaMK1*β* | 25.13 | 21.81 | 2.35 | 23.47 |
| PKC*α* | 18.34 | 28.55 | 7.23 | 23.44 |
| EGFR L861Q | 26.25 | 20.02 | 4.41 | 23.14 |
| PRKAR1A-RET | 25.38 | 20.58 | 3.40 | 22.98 |
| ERN1 | 20.44 | 25.11 | 3.30 | 22.77 |
| IRAK1 | 27.61 | 17.50 | 7.15 | 22.56 |
| CK1*ε* | 24.96 | 19.87 | 3.60 | 22.41 |
| NEK2 | 14.96 | 28.87 | 9.84 | 21.92 |
| PHKG2 | 21.14 | 22.49 | 0.95 | 21.82 |
| PKD3 | 25.53 | 18.08 | 5.27 | 21.80 |
| AMPK*α*1/*β*1/*γ*1 | 27.26 | 14.10 | 9.30 | 20.68 |
| PDGFR*α* T674I | 12.71 | 27.99 | 10.81 | 20.35 |
| CaMK2*δ* | 16.09 | 23.16 | 5.00 | 19.63 |
| HIPK1 | 9.14 | 30.04 | 14.78 | 19.59 |
| TGF*β*R1 | 20.86 | 18.04 | 1.99 | 19.45 |
| MET D1228H | 22.66 | 15.98 | 4.73 | 19.32 |
| TRKB | 14.62 | 23.50 | 6.28 | 19.06 |
| CK1*γ*3 | 19.43 | 18.35 | 0.76 | 18.89 |
| PKR | 9.34 | 27.01 | 12.50 | 18.18 |
| PRKX | 23.37 | 12.80 | 7.48 | 18.08 |
| AKT2 | 10.39 | 25.39 | 10.61 | 17.89 |
| EGFR D746-750 T790M C797S L858R | 18.72 | 17.05 | 1.18 | 17.88 |
| MET | 16.80 | 18.59 | 1.27 | 17.70 |
| MELK | 16.23 | 18.46 | 1.58 | 17.34 |
| PIK3CD | 21.16 | 12.81 | 5.90 | 16.99 |
| PAK5 | 14.52 | 19.36 | 3.42 | 16.94 |
| LOK | 18.20 | 15.68 | 1.78 | 16.94 |
| MST2 | 19.21 | 14.67 | 3.21 | 16.94 |
| TLK1 | 4.66 | 29.03 | 17.24 | 16.84 |
| CDK2/CycA2 | 15.25 | 17.28 | 1.44 | 16.27 |
| MLK1 | 17.04 | 15.21 | 1.29 | 16.13 |
| TGF*β*R1 T204D | 2.59 | 29.45 | 18.99 | 16.02 |
| KIT D816V | 20.72 | 11.22 | 6.72 | 15.97 |
| AKT1 | 12.74 | 19.00 | 4.43 | 15.87 |
| DYRK3 | 7.74 | 23.28 | 10.99 | 15.51 |
| CDK3/CycE1 | 17.25 | 12.44 | 3.40 | 14.85 |
| CDK2/CycE1 | 18.33 | 11.14 | 5.08 | 14.73 |
| PLK3 | 6.39 | 22.72 | 11.54 | 14.56 |
| EGFR d746-750 T790M C797S | 18.13 | 10.92 | 5.10 | 14.52 |
| AMPK*α*2/*β*1/*γ*1 | 16.82 | 11.69 | 3.62 | 14.26 |
| p70S6K | 16.14 | 12.33 | 2.70 | 14.23 |
| DCAMKL2 | 17.49 | 10.90 | 4.66 | 14.20 |
| RET R921P | 20.65 | 7.72 | 9.14 | 14.19 |
| CAMKK2 | 16.74 | 11.16 | 3.95 | 13.95 |
| NIM1K | 14.69 | 12.95 | 1.23 | 13.82 |
| FLT1 | 21.36 | 6.07 | 10.81 | 13.71 |
| CHK1 | 8.63 | 18.75 | 7.16 | 13.69 |
| TRKA G667A | 18.30 | 8.22 | 7.13 | 13.26 |
| MARK1 | 15.11 | 10.77 | 3.07 | 12.94 |
| RSK1 | 9.69 | 16.01 | 4.47 | 12.85 |
| ALK2 | 2.60 | 22.76 | 14.25 | 12.68 |
| AKT3 | 2.57 | 22.47 | 14.07 | 12.52 |
| GSK3*β* | 14.76 | 10.26 | 3.18 | 12.51 |
| CaMK2*β* | 15.13 | 9.88 | 3.71 | 12.51 |
| PRKCE | 10.26 | 14.68 | 3.13 | 12.47 |
| MUSK | 1.67 | 23.04 | 15.11 | 12.36 |
| AMPK*α*2/*β*2/*γ*3 | 3.96 | 20.62 | 11.78 | 12.29 |
| PKN3 | 4.94 | 19.15 | 10.04 | 12.04 |
| p38*β* | 11.48 | 12.34 | 0.61 | 11.91 |
| CSK | 12.26 | 11.08 | 0.83 | 11.67 |
| FGFR3 V555L | 15.52 | 7.24 | 5.85 | 11.38 |
| RET G810R | 6.36 | 16.36 | 7.07 | 11.36 |
| EGFR T790M C797S | 19.29 | 2.47 | 11.89 | 10.88 |
| PAK1 | 6.40 | 15.26 | 6.27 | 10.83 |
| MET Y1230D | 12.73 | 8.72 | 2.84 | 10.73 |
| SRM | 11.23 | 10.19 | 0.74 | 10.71 |
| RET M918T | 16.74 | 4.58 | 8.60 | 10.66 |
| PKAC*α* | 11.29 | 10.03 | 0.89 | 10.66 |
| ROS1 | 7.67 | 12.80 | 3.63 | 10.23 |
| p38*δ* | 15.59 | 4.71 | 7.70 | 10.15 |
| FGFR3 K650E | 15.54 | 4.42 | 7.87 | 9.98 |
| ULK2 | 19.54 | 0.28 | 13.62 | 9.91 |
| AMPK*α*2/*β*2/*γ*1 | 11.45 | 8.35 | 2.19 | 9.90 |
| YES | 1.07 | 18.50 | 12.32 | 9.79 |
| MAP3K5 | 1.99 | 17.02 | 10.63 | 9.51 |
| JAK2 | 20.13 | -1.29 | 15.15 | 9.42 |
| MLK3 | 14.95 | 3.72 | 7.94 | 9.33 |
| MST3 | 9.23 | 9.25 | 0.02 | 9.24 |
| SLK | 4.49 | 13.61 | 6.45 | 9.05 |
| NEK1 | 11.46 | 6.49 | 3.52 | 8.98 |
| BARK2 | 7.48 | 10.29 | 1.99 | 8.89 |
| MAP2K7 | 4.28 | 13.43 | 6.47 | 8.85 |
| FGFR2 R612T | 14.20 | 3.40 | 7.64 | 8.80 |
| FGFR10P-FGFR | 5.53 | 11.99 | 4.57 | 8.76 |
| MAP3K2 | 12.39 | 5.14 | 5.12 | 8.76 |
| AMPK*α*2/*β*1/*γ*2 | 8.00 | 9.34 | 0.95 | 8.67 |
| BMPR2 | 1.30 | 16.03 | 10.41 | 8.66 |
| WNK1 | 13.58 | 3.73 | 6.96 | 8.66 |
| MAP3K3 | 7.03 | 10.26 | 2.28 | 8.65 |
| CK1*α* | 4.01 | 12.85 | 6.25 | 8.43 |
| RSK3 | 11.25 | 5.60 | 3.99 | 8.42 |
| PRKCQ | 12.48 | 4.16 | 5.89 | 8.32 |
| ALK | 4.11 | 12.24 | 5.75 | 8.17 |
| PRKCH | 10.69 | 5.61 | 3.59 | 8.15 |
| EGFR L858R T790M | 16.03 | 0.16 | 11.22 | 8.09 |
| MRCK*β* | 8.86 | 7.26 | 1.13 | 8.06 |
| PAK3 | 7.83 | 8.08 | 0.18 | 7.96 |
| GAK | 15.56 | 0.19 | 10.87 | 7.88 |
| CaMK2*α* | 7.27 | 7.92 | 0.46 | 7.59 |
| LATS2 | 10.59 | 4.58 | 4.25 | 7.59 |
| IKK*β* | 4.35 | 10.82 | 4.58 | 7.58 |
| RSK4 | 4.63 | 10.52 | 4.17 | 7.57 |
| RSK2 | 4.84 | 10.25 | 3.82 | 7.54 |
| PKAC*β* | 8.38 | 6.56 | 1.29 | 7.47 |
| NuaK2 | 8.49 | 6.43 | 1.46 | 7.46 |
| GLK | 9.63 | 4.89 | 3.35 | 7.26 |
| FGFR4 | 5.45 | 8.55 | 2.19 | 7.00 |
| EPHA8 | -2.75 | 16.71 | 13.76 | 6.98 |
| OSR1 | 8.92 | 4.99 | 2.78 | 6.95 |
| MAP3K4 | 13.42 | 0.36 | 9.23 | 6.89 |
| SIK3 | 6.05 | 7.61 | 1.11 | 6.83 |
| NDR1 | -0.28 | 13.41 | 9.68 | 6.56 |
| PAK2 | 5.86 | 7.09 | 0.86 | 6.48 |
| NCOA4-RET | 2.24 | 10.61 | 5.92 | 6.42 |
| CHK2 | -3.43 | 16.21 | 13.89 | 6.39 |
| FGFR4 V550M | 4.40 | 8.07 | 2.60 | 6.24 |
| CaMK1*α* | -0.58 | 12.98 | 9.59 | 6.20 |
| HIPK4 | 3.70 | 8.62 | 3.48 | 6.16 |
| MAP3K19 | 15.33 | -3.86 | 13.57 | 5.74 |
| CK2*α*2/*β* | 1.94 | 9.50 | 5.35 | 5.72 |
| DYRK4 | -5.86 | 17.03 | 16.19 | 5.58 |
| FLT4 | 6.76 | 4.30 | 1.74 | 5.53 |
| WEE1 | 2.48 | 8.54 | 4.29 | 5.51 |
| PIK3CB | 5.55 | 5.39 | 0.11 | 5.47 |
| MNK2 | 2.50 | 8.36 | 4.15 | 5.43 |
| CRAF | 13.00 | -2.16 | 10.72 | 5.42 |
| CCDC6-RET | 9.08 | 1.57 | 5.31 | 5.32 |
| GSK3*α* | 6.00 | 4.43 | 1.11 | 5.21 |
| MST4 | -1.76 | 12.17 | 9.85 | 5.21 |
| TEC | 7.53 | 2.70 | 3.41 | 5.12 |
| FGFR2 | 3.38 | 6.82 | 2.43 | 5.10 |
| FER | 10.07 | -0.50 | 7.48 | 4.79 |
| TBK1 | -0.45 | 9.89 | 7.31 | 4.72 |
| EPHA5 | 0.67 | 8.74 | 5.70 | 4.70 |
| FGFR1 | -0.31 | 9.70 | 7.08 | 4.70 |
| RET V804L | 11.07 | -1.73 | 9.05 | 4.67 |
| AurC | 15.05 | -5.92 | 14.83 | 4.56 |
| AMPK*α*1/*β*1/*γ*2 | 1.36 | 7.71 | 4.49 | 4.54 |
| SIK | 4.66 | 4.38 | 0.20 | 4.52 |
| PKN1 | 6.67 | 2.06 | 3.26 | 4.36 |
| WNK3 | 7.91 | 0.62 | 5.15 | 4.27 |
| MARK2 | 4.73 | 3.76 | 0.69 | 4.25 |
| GPRK4 | 1.14 | 7.33 | 4.37 | 4.23 |
| LRRK2 G2019S | 13.84 | -5.39 | 13.60 | 4.22 |
| PLK1 | 5.82 | 2.45 | 2.38 | 4.14 |
| LIMK1 | 2.35 | 5.89 | 2.50 | 4.12 |
| CDK1/CycB1 | -4.84 | 13.03 | 12.64 | 4.10 |
| NPM1-ALK | 15.27 | -7.09 | 15.81 | 4.09 |
| NDR2 | -2.37 | 10.54 | 9.13 | 4.09 |
| EPHA7 | 2.45 | 5.56 | 2.19 | 4.00 |
| TRKA G595R G667A | 12.33 | -4.47 | 11.88 | 3.93 |
| FGFR3 | -3.09 | 10.79 | 9.81 | 3.85 |
| MRCK*α* | 4.74 | 2.93 | 1.28 | 3.83 |
| ROCK2 | 7.56 | 0.07 | 5.30 | 3.81 |
| HPK1 | 6.19 | 1.42 | 3.37 | 3.81 |
| ULK3 | 0.98 | 6.59 | 3.97 | 3.79 |
| EGFR T790M | 18.51 | -10.95 | 20.83 | 3.78 |
| MLK2 | 12.74 | -5.57 | 12.95 | 3.58 |
| FLT3 D835Y | 10.39 | -3.24 | 9.64 | 3.57 |
| RET G691S | 4.43 | 2.53 | 1.35 | 3.48 |
| MARK4 | 3.68 | 3.28 | 0.28 | 3.48 |
| CK2*α*1/*β* | 8.65 | -1.71 | 7.32 | 3.47 |
| EGFR L858R | 8.99 | -2.05 | 7.81 | 3.47 |
| IGF1R | 13.43 | -6.65 | 14.20 | 3.39 |
| CRIK | 3.60 | 3.12 | 0.34 | 3.36 |
| TRKC G623E | 6.27 | 0.41 | 4.14 | 3.34 |
| SRC | -6.55 | 13.20 | 13.96 | 3.32 |
| EGFR C797S | 6.05 | 0.57 | 3.87 | 3.31 |
| FES | 10.39 | -3.85 | 10.07 | 3.27 |
| RET | 0.75 | 5.58 | 3.42 | 3.16 |
| LYNb | -1.18 | 7.50 | 6.14 | 3.16 |
| CaMK1*δ* | -4.06 | 10.28 | 10.14 | 3.11 |
| AAK1 | 2.64 | 3.48 | 0.59 | 3.06 |
| PASK | -1.54 | 7.58 | 6.45 | 3.02 |
| LATS1 | 1.04 | 4.96 | 2.77 | 3.00 |
| FGFR2 C491S | 2.52 | 3.14 | 0.43 | 2.83 |
| TRKA | -5.28 | 10.85 | 11.41 | 2.79 |
| DDR2 | -2.07 | 7.58 | 6.82 | 2.75 |
| p38*γ* | 0.39 | 5.05 | 3.30 | 2.72 |
| FYN [isoform b] | 4.46 | 0.90 | 2.52 | 2.68 |
| TRKC L686M | -4.12 | 9.37 | 9.54 | 2.62 |
| PYK2 | 0.74 | 4.47 | 2.63 | 2.61 |
| MSK1 | 2.72 | 2.30 | 0.30 | 2.51 |
| TNIK | 1.50 | 3.25 | 1.24 | 2.38 |
| FRK | 5.85 | -1.13 | 4.93 | 2.36 |
| PKC*β*1 | -3.17 | 7.83 | 7.78 | 2.33 |
| PLK4 | 1.16 | 3.49 | 1.64 | 2.33 |
| EGFR D770_N771insNPG | 4.21 | 0.43 | 2.67 | 2.32 |
| P38*α* | 3.85 | 0.56 | 2.32 | 2.21 |
| FGFR2 E565G | 4.72 | -0.33 | 3.57 | 2.19 |
| TSSK3 | -0.18 | 4.57 | 3.36 | 2.19 |
| ROCK1 | -2.43 | 6.66 | 6.42 | 2.12 |
| TSSK1 | -1.19 | 5.42 | 4.68 | 2.11 |
| PGK | 4.06 | 0.08 | 2.81 | 2.07 |
| EPHA3 | 13.85 | -9.90 | 16.79 | 1.97 |
| FLT3 ITD | 7.44 | -3.64 | 7.83 | 1.90 |
| CaMK2*γ* | 4.34 | -0.57 | 3.47 | 1.88 |
| LYNa | 5.62 | -1.89 | 5.31 | 1.87 |
| FGFR2 V564I | 1.71 | 1.99 | 0.20 | 1.85 |
| WNK2 | 8.84 | -5.14 | 9.89 | 1.85 |
| PDGFR*α* V561D | -12.50 | 16.09 | 20.22 | 1.80 |
| AMPK*α*1/*β*1/*γ*3 | 6.06 | -2.48 | 6.04 | 1.79 |
| TRKA A608D | 11.28 | -7.78 | 13.48 | 1.75 |
| PRKCI | 5.17 | -1.78 | 4.92 | 1.69 |
| IRR | -4.86 | 8.09 | 9.16 | 1.61 |
| NEK9 | 0.49 | 2.52 | 1.43 | 1.51 |
| CLK4 | -0.48 | 3.46 | 2.79 | 1.49 |
| BRAF | -0.42 | 3.37 | 2.68 | 1.47 |
| TRKA G595R | 5.54 | -3.14 | 6.14 | 1.20 |
| RET S904A | 2.36 | -0.52 | 2.03 | 0.92 |
| PAK4 | 0.65 | 0.88 | 0.17 | 0.77 |
| CSF1R | -6.02 | 7.52 | 9.57 | 0.75 |
| EGFR A763_Y764insFQEA | 3.41 | -1.92 | 3.77 | 0.74 |
| TRKC | 0.63 | 0.73 | 0.07 | 0.68 |
| ErK1 | -1.06 | 1.75 | 1.99 | 0.34 |
| AMPK*α*1/*β*2/*γ*1 | -2.57 | 3.17 | 4.06 | 0.30 |
| ITK | -3.79 | 4.25 | 5.68 | 0.23 |
| RET L730I | 6.37 | -5.91 | 8.69 | 0.23 |
| HIPK2 | -6.41 | 6.85 | 9.37 | 0.22 |
| NLK | -5.96 | 6.36 | 8.71 | 0.20 |
| EPHA2 | -4.31 | 4.71 | 6.38 | 0.20 |
| CLK2 | 0.06 | 0.22 | 0.12 | 0.14 |
| STK33 | -3.62 | 3.78 | 5.23 | 0.08 |
| PDGFR*α* | -3.98 | 4.11 | 5.72 | 0.07 |
| EPHB3 | -5.14 | 5.17 | 7.30 | 0.01 |
| EGFR d746-750 | -14.01 | 13.67 | 19.58 | -0.17 |
| RIPK2 | -2.54 | 1.86 | 3.11 | -0.34 |
| EGFR | -8.57 | 7.77 | 11.56 | -0.40 |
| NuaK1 | 0.37 | -1.32 | 1.19 | -0.47 |
| AMPK*α*1/*β*2/*γ*2 | 0.34 | -1.53 | 1.32 | -0.59 |
| FGFR2 K659N | 1.64 | -2.89 | 3.20 | -0.63 |
| GPRK7 | -10.26 | 8.94 | 13.57 | -0.66 |
| ZAK | 9.87 | -11.28 | 14.95 | -0.71 |
| TRKA G667S | 12.58 | -14.09 | 18.86 | -0.75 |
| BTK T316A | -11.34 | 9.64 | 14.84 | -0.85 |
| FGFR2 N549H | -3.15 | 1.40 | 3.22 | -0.87 |
| RON | -4.22 | 2.17 | 4.52 | -1.03 |
| EGFR d746-750 T790M | -6.54 | 4.22 | 7.61 | -1.16 |
| FGFR3 G697C | -2.75 | 0.32 | 2.17 | -1.22 |
| CLK3 | -4.33 | 1.79 | 4.33 | -1.27 |
| RET V804E | -8.16 | 5.54 | 9.68 | -1.31 |
| TTBK1 | -2.90 | 0.19 | 2.18 | -1.35 |
| DCAMKL1 | -9.50 | 6.08 | 11.01 | -1.71 |
| FGFR3 K650Q | 5.90 | -9.33 | 10.77 | -1.72 |
| FGFR3 K650M | -3.37 | -0.18 | 2.25 | -1.78 |
| RET S904F | -3.65 | 0.08 | 2.63 | -1.79 |
| KDR | 4.38 | -8.14 | 8.86 | -1.88 |
| ULK1 | -1.21 | -2.62 | 1.00 | -1.91 |
| IKK*α* | -6.88 | 2.88 | 6.90 | -2.00 |
| BTK T474I | -2.75 | -1.25 | 1.06 | -2.00 |
| MAPKAPK3 | 0.19 | -4.81 | 3.54 | -2.31 |
| ABL1 H396P | -3.05 | -1.57 | 1.04 | -2.31 |
| BTK C481S | -8.93 | 4.04 | 9.17 | -2.44 |
| TTBK2 | -8.54 | 3.60 | 8.59 | -2.47 |
| ZAP70 | -4.90 | -0.15 | 3.36 | -2.53 |
| BUB1/BUB3 | -4.07 | -1.07 | 2.12 | -2.57 |
| PIM2 | 4.66 | -9.90 | 10.30 | -2.62 |
| FGFR2 C491F | -2.37 | -3.08 | 0.50 | -2.72 |
| RET Y806H | -0.97 | -4.49 | 2.49 | -2.73 |
| EPHB1 | -7.85 | 2.29 | 7.17 | -2.78 |
| FLT3 | -4.31 | -1.28 | 2.14 | -2.79 |
| RET V804M | -2.33 | -3.41 | 0.76 | -2.87 |
| EGFR d746-750 C797G | 8.49 | -14.63 | 16.35 | -3.07 |
| EPHA4 | -12.29 | 6.03 | 12.96 | -3.13 |
| AMPK*α*2/*β*2/*γ*2 | -7.17 | 0.78 | 5.63 | -3.20 |
| TRKC G696A | 1.74 | -8.39 | 7.17 | -3.32 |
| PIM1 | -5.05 | -1.74 | 2.34 | -3.39 |
| AurB | 0.28 | -7.07 | 5.20 | -3.40 |
| KIT | -12.85 | 5.67 | 13.10 | -3.59 |
| FGFR4 V550L | -3.73 | -3.51 | 0.16 | -3.62 |
| DMPK1 | 0.78 | -8.20 | 6.35 | -3.71 |
| PAK6 | -7.99 | 0.50 | 6.00 | -3.75 |
| DAPK1 | -11.10 | 3.13 | 10.06 | -3.98 |
| MST1 | -10.21 | 1.64 | 8.38 | -4.28 |
| FGFR2 K641R | -3.44 | -5.42 | 1.40 | -4.43 |
| BRSK1 | 0.06 | -9.25 | 6.58 | -4.59 |
| IRAK4 | -4.82 | -4.50 | 0.23 | -4.66 |
| PIM3 | -15.30 | 5.98 | 15.05 | -4.66 |
| FGFR2 V564F | 0.44 | -10.04 | 7.42 | -4.80 |
| TRKA G667C | -1.11 | -8.51 | 5.23 | -4.81 |
| PRKCD | -7.61 | -2.45 | 3.65 | -5.03 |
| EGFR V769-D770 insGE | 5.48 | -15.56 | 14.88 | -5.04 |
| NEK4 | -4.06 | -6.43 | 1.68 | -5.25 |
| MINK | -3.21 | -7.49 | 3.03 | -5.35 |
| MAPKAPK5 | -1.66 | -9.13 | 5.28 | -5.39 |
| HIPK3 | -9.63 | -1.41 | 5.82 | -5.52 |
| HER4 | -13.44 | 2.40 | 11.20 | -5.52 |
| ACK | -3.68 | -7.78 | 2.90 | -5.73 |
| TLK2 | -9.53 | -1.98 | 5.34 | -5.75 |
| DMPK2 | -4.50 | -7.12 | 1.86 | -5.81 |
| RET L790F | -10.87 | -1.54 | 6.60 | -6.21 |
| MARK3 | -14.05 | 1.60 | 11.06 | -6.23 |
| FGFR3 V555M | -4.50 | -8.22 | 2.63 | -6.36 |
| MNK1 | -8.83 | -3.92 | 3.47 | -6.37 |
| MAP3K1 | -9.73 | -3.09 | 4.69 | -6.41 |
| EGFR d746-750 C797S | -6.27 | -7.07 | 0.56 | -6.67 |
| EPHA1 | -6.16 | -7.57 | 1.00 | -6.86 |
| ABL1 | -11.97 | -1.80 | 7.19 | -6.89 |
| MAP4K2 | -9.71 | -4.82 | 3.46 | -7.27 |
| TRKA G595R A608D | -9.02 | -5.66 | 2.38 | -7.34 |
| AMPK*α*2/*β*1/*γ*3 | -5.91 | -9.22 | 2.34 | -7.57 |
| MYO3*β* | -4.60 | -11.30 | 4.74 | -7.95 |
| RET L730M | -6.47 | -9.52 | 2.16 | -8.00 |
| IKK*ε* | -6.35 | -9.76 | 2.41 | -8.05 |
| BTK | -13.87 | -2.52 | 8.02 | -8.19 |
| GPRK6 | -10.56 | -6.05 | 3.19 | -8.31 |
| KHS | -13.41 | -3.35 | 7.11 | -8.38 |
| MSK2 | -9.72 | -7.12 | 1.84 | -8.42 |
| MAP2K5 | -10.45 | -6.52 | 2.78 | -8.49 |
| FGFR4 N535K | -6.30 | -11.12 | 3.41 | -8.71 |
| MAP2K2 | -15.01 | -2.42 | 8.90 | -8.72 |
| EPHB4 | -17.39 | -0.38 | 12.03 | -8.89 |
| PDGFR*β* | -15.21 | -2.61 | 8.91 | -8.91 |
| MAP2K3 | -6.52 | -11.30 | 3.38 | -8.91 |
| EGFR T790M C797S L858R | -12.89 | -4.93 | 5.63 | -8.91 |
| FYN [isoform a] | -4.73 | -13.40 | 6.12 | -9.07 |
| QIK | -4.62 | -13.52 | 6.30 | -9.07 |
| BRK | -6.89 | -11.30 | 3.12 | -9.10 |
| JAK3 | -13.65 | -4.66 | 6.36 | -9.15 |
| PIK3CA | 3.44 | -21.97 | 17.97 | -9.26 |
| GSG2 | -16.82 | -1.76 | 10.65 | -9.29 |
| SYK | -13.91 | -5.04 | 6.27 | -9.48 |
| COT | -14.24 | -5.12 | 6.45 | -9.68 |
| Erk2 | -17.67 | -1.86 | 11.18 | -9.77 |
| BRAF V600E | -3.80 | -16.17 | 8.74 | -9.98 |
| AXL | -6.47 | -13.51 | 4.98 | -9.99 |
| TPR-TRKA | -7.05 | -14.08 | 4.97 | -10.57 |
| TFG-TRKA | -11.79 | -10.02 | 1.25 | -10.91 |
| CDC7/ASK | -14.78 | -7.04 | 5.47 | -10.91 |
| LCK | -15.15 | -7.76 | 5.23 | -11.46 |
| KIT V559D | -1.88 | -21.15 | 13.63 | -11.52 |
| FGFR2 K526E | 1.54 | -25.28 | 18.96 | -11.87 |
| TIE2 | -8.86 | -15.46 | 4.66 | -12.16 |
| ALK4 | -11.17 | -14.01 | 2.01 | -12.59 |
| KIT T670I | -13.05 | -12.57 | 0.34 | -12.81 |
| PEK | -0.75 | -24.92 | 17.09 | -12.83 |
| CLK1 | -15.27 | -10.76 | 3.19 | -13.01 |
| BRSK2 | -14.64 | -11.56 | 2.18 | -13.10 |
| TYK2 | -13.77 | -13.10 | 0.48 | -13.43 |
| FAK | -24.19 | -2.77 | 15.15 | -13.48 |
| MAPKAPK2 | -5.03 | -22.68 | 12.48 | -13.85 |
| BMX | -4.57 | -23.23 | 13.19 | -13.90 |
| TYRO3 | -13.18 | -14.87 | 1.20 | -14.02 |
| EGFR C797S L858R | -7.13 | -20.94 | 9.76 | -14.03 |
| HCK | -8.72 | -19.95 | 7.95 | -14.33 |
| HER2 | -22.79 | -7.01 | 11.16 | -14.90 |
| HGK | -10.65 | -19.37 | 6.17 | -15.01 |
| P70S6K*β* | -18.98 | -11.08 | 5.59 | -15.03 |
| MAP2K1 | -17.03 | -13.22 | 2.69 | -15.13 |
| PKAC*γ* | -6.62 | -23.92 | 12.23 | -15.27 |
| INSR | -16.49 | -14.08 | 1.71 | -15.28 |
| EGFR D746-750 T790M L858R | -23.35 | -7.23 | 11.40 | -15.29 |
| FGR | -13.61 | -17.58 | 2.81 | -15.60 |
| PBK | -18.92 | -12.36 | 4.64 | -15.64 |
| ABL2 | -25.54 | -6.04 | 13.78 | -15.79 |
| JAK1 | -7.07 | -25.04 | 12.71 | -16.06 |
| BARK1 | -25.86 | -7.05 | 13.30 | -16.46 |
| TGF*β*R2 | -29.51 | -3.63 | 18.30 | -16.57 |
| TXK | -17.18 | -16.16 | 0.72 | -16.67 |
| RET E762Q | -10.03 | -25.60 | 11.01 | -17.81 |
| NEK3 | -15.85 | -19.94 | 2.89 | -17.89 |
| AurA | -22.00 | -15.64 | 4.50 | -18.82 |
| EPHA6 | -12.66 | -25.70 | 9.22 | -19.18 |
| BLK | -30.18 | -10.58 | 13.86 | -20.38 |
| AMPK*α*1/*β*2/*γ*3 | -21.11 | -20.13 | 0.69 | -20.62 |
| EPHB2 | -20.23 | -21.87 | 1.16 | -21.05 |
| PLK2 | -13.96 | -30.43 | 11.65 | -22.20 |
| NIK | -25.30 | -20.44 | 3.44 | -22.87 |
| TNK1 | -25.39 | -25.65 | 0.19 | -25.52 |
| BTK T474S | -19.29 | -32.06 | 9.03 | -25.68 |
| YSK1 | -28.15 | -27.62 | 0.38 | -27.88 |

**Table S3** Results of CYP Phenotyping Screening of ZLC853

| Tested cpd | Preincubation of inhibitor  for corresponding CYP | Percent remaining (%) | Inhibition ratio (%) |
| --- | --- | --- | --- |
| **ZLC853** | T_0_ | 100 | – |
|  | No inhibitor | 46.8 | – |
|  | Furafylline for 1A2 | 48.2 | 2.69 |
|  | Ticlopidine for 2B6 | 45.4 | –2.56 |
|  | Montelukast for 2C8 | 57.5 | 20.2 |
|  | Sulfaphpenazole for 2C9 | 55.3 | 16.1 |
|  | N-3-benzylnirvanol for 2C19 | 50.7 | 7.42 |
|  | Quinidine for 2D6 | 49.1 | 4.48 |
|  | Ketoconazole for 3A | 93.9 | 88.5 |

**Table S4** Primers Used in RT-qPCR

| Primers | Sequences (5′ to 3′) |
| --- | --- |
| *ATM*-F | GCTGACAATCATCACCAAGT |
| *ATM*-R | GGTTCTCAGCACTATGGGACA |
| *ATR*-F | CGCTGAACTGTACGTGGAAA |
| *ATR*-R | CAATTAGTGCCTGGTGAACATC |
| *BRCA1*-F | CTGCTCAGGGCTATCCTCTCA |
| *BRCA1*-R | GCTTCTAGTTCAGCCATTTCCTG |
| *FANCI*-F | CACCACACTTACAGCCCTTG |
| *FANCI*-R | ATTCCTCCGGAGCTCTGAC |
| *RAD51*-F | GCTGATGAGTTTGGTGTAGCAG |
| *RAD51*-R | GGAAGACAGGGAGAGTCGTAGA |
| *ACTIN*-F | AGGATGCAGAAGGAGATCACTG |
| *ACTIN*-R | AGTACTTGCGCTCAGGAGGAG |
| *NRAS*-F | GCGAAGGCTTCCTCTGTGTA |
| *NRAS*-R | CTTGTTTCCCACTAGCACCA |

**The ^1^H NMR, ^13^C NMR, HRMS and HPLC traces of Compounds 1a-d, ZLC1178 and ZLC1223.**
